# Efficacy of biologically-directed daylight therapy on sleep and circadian rhythm in Parkinson's disease: a randomised, double-blind, parallel-group, active-controlled, phase 2 clinical trial

**DOI:** 10.1016/j.eclinm.2024.102474

**Published:** 2024-02-10

**Authors:** Beatrix Feigl, Simon J.G. Lewis, Lucy D. Burr, Daniel Schweitzer, Subodh Gnyawali, Dimitrios Vagenas, Drew D. Carter, Andrew J. Zele

**Affiliations:** a Centre for Vision and Eye Research, Queensland University of Technology (QUT), Brisbane, QLD, 4059, Australia; b School of Biomedical Sciences, Queensland University of Technology (QUT), Brisbane, QLD, 4059, Australia; c Queensland Eye Institute, South Brisbane, QLD, 4101, Australia; d Brain and Mind Centre, The University of Sydney, New South Wales, 2006, Australia; e Department of Respiratory and Sleep Medicine, Mater Health, South Brisbane, QLD, 4101, Australia; f Mater Research, University of Queensland, QLD, 4072, Australia; g Centre of Neurosciences, Mater Health, South Brisbane, QLD, 4101, Australia; h Wesley Hospital, Auchenflower, QLD, 4066, Australia; i School of Public Health and Social Work, Queensland University of Technology (QUT), Brisbane, QLD, 4059, Australia

**Keywords:** Melanopsin, Light, Sleep, Polysomnography, Motor

## Abstract

**Background:**

New non-pharmacological treatments for improving non-motor symptoms in Parkinson's disease (PD) are urgently needed. Previous light therapies for modifying sleep behaviour lacked standardised protocols and were not personalised for an individual patient chronotype. We aimed to assess the efficacy of a biologically-directed light therapy in PD that targets retinal inputs to the circadian system on sleep, as well as other non-motor and motor functions.

**Methods:**

In this randomised, double-blind, parallel-group, active-controlled trial at the Queensland University of Technology, Australia, participants with mild to moderate PD were computer randomised (1:1) to receive one of two light therapies that had the same photometric luminance and visual appearance to allow blinding of investigators and participants to the intervention. One of these biologically-directed lights matched natural daylight (Day Mel), which is known to stimulate melanopsin cells. The light therapy of the other treatment arm of the study, specifically supplemented the stimulation of retinal melanopsin cells (Enhanced Mel), targeting deficits to the circadian system. Both lights were administered 30 min per day over 4-weeks and personalised to an individual patient's chronotype, while monitoring environmental light exposure with actigraphy. Co-primary endpoints were a change from baseline in mean sleep macrostructure (polysomnography, PSG) and an endocrine biomarker of circadian phase (dim light melatonin secretion onset, DLMO) at weeks 4 and 6. Participants data were analysed using an intention to treat principle. All endpoints were evaluated by applying a mixed model analysis. The trial is registered with the Australian New Zealand Clinical Trials Registry, ACTRN12621000077864.

**Findings:**

Between February 4, 2021 and August 8, 2022, 144 participants with PD were consecutively screened, 60 enrolled and randomly assigned to a light intervention. There was no significant difference in co-primary outcomes between randomised groups overall or at any individual timepoint during follow-up. The mean (95% CI) for PSG, N3% was 24.15 (19.82–28.48) for Day Mel (n = 23) and 19.34 (15.20–23.47) for the Enhanced Mel group (n = 25) in week 4 (p = 0.12); and 21.13 (16.99–25.28) for Day Mel (n = 26) and 18.48 (14.34–22.62) for the Enhanced Mel group (n = 25) in week 6, (p = 0.37). The mean (95% CI) DLMO (decimal time) was 19.82 (19.20–20.44) for Day Mel (n = 22) and 19.44 (18.85–20.04) for the Enhanced Mel group (n = 24) in week 4 (p = 0.38); and 19.90 (19.27–20.53) for Day Mel (n = 23) and 19.04 (18.44–19.64) for the Enhanced Mel group (n = 25) in week 6 (p = 0.05). However, both the controlled daylight (Day Mel) and the enhanced melanopsin (Enhanced Mel) interventions demonstrated significant improvement in primary PSG sleep macrostructure. The restorative deep sleep phase (PSG, N3) significantly improved at week 6 in both groups [model-based mean difference to baseline (95% CI): −3.87 (−6.91 to −0.83), p = 0.04]. There was a phase-advance in DLMO in both groups which did not reach statistical significance between groups at any time-point. There were no safety concerns or severe adverse events related to the intervention.

**Interpretation:**

Both the controlled daylight and melanopsin booster light showed efficacy in improving measures of restorative deep sleep in people with mild to moderate PD. That there was no significant difference between the two intervention groups may be due to the early disease stage. The findings suggest that controlled indoor daylight that is personalised to the individuals’ chronotype could be effective for improving sleep in early to moderate PD, and further studies evaluating controlled daylight interventions are now required utilising this standardised approach, including in advanced PD.

**Funding:**

The 10.13039/100000864Michael J Fox Foundation for Parkinson’s Research, Shake IT Up Australia, 10.13039/501100000925National Health and Medical Research Council, and 10.13039/501100000923Australian Research Council.

**Research in context**

**Evidence before this study**

We searched the National Library of Medicine database using the terms “light therapy” and “Parkinson's disease”, “randomized controlled”, “non-motor symptoms” and “melanopsin”, with no date parameters or cut off. We conducted a literature search between 12/2019 and 12/2023. The search revealed several findings including that: (1) the development and testing of new treatments for non-motor symptoms in Parkinson's disease (PD) remain a top priority; (2) the effects of light therapy in alleviating non-motor (including sleep) and motor symptoms in PD are inconclusive; (3) there is no standard light intervention, and none that match the biological effects of natural sunlight; and (4) light intervention studies should be personalised and mechanism based to target the pathology of melanopsin retinal inputs to the retinohypothalamic tract that contribute to the circadian disruption, including sleep abnormalities.

**Added value of this study**

This randomized, double-blind, active-controlled clinical trial in people with PD evaluated a light intervention that matched the biological effect of natural daylight on melanopsin cells (Day Mel) and another with the same luminance and colour appearance that specifically targeted the dysfunctional melanopsin cell activity (Enhanced Mel). Furthermore, we personalised the timing of both light interventions based on a patient's individual chronotype and also monitored the ambient light levels to exclude the impact of additional environmental light exposure on the outcomes. To date this study represents the most comprehensive investigation of light therapy in PD. Our findings suggest that both, Day Mel and Enhanced Mel therapies may be effective in alleviating non-motor symptoms, with no statistically significant difference between their benefits, although there was a trend towards the enhanced light condition. In addition to improving restorative deep sleep, this study also reports better wellbeing and motor function in patients receiving personalised light therapy based on chronotype, as well as caregivers rated improvements in sleep quality.

**Implications of all the available evidence**

This RCT provides early evidence that a personalised, biologically-directed daylight therapy may improve sleep in patients with PD, and further research is needed to examine the role of light therapy on sleep and other non-motor and motor functions. We recommend to use a controlled daylight interventions targeting retinohypothalamic tract abnormalities as a standard in subsequent clinical trials.

## Introduction

Individuals with Parkinson's disease (PD) frequently experience circadian and sleep-related disorders,^1^ but pharmacological treatments have had limited success, often causing side effects and/or aggravating clinical symptoms.^2^ It is the current view that the therapeutic effect of light therapy on sleep in PD is mediated via the circadian system and likely due to synchronization of the circadian clock.^3^^,^^4^ While light therapy is an effective non-invasive photoceutical alternative in depressive disorders, two recent meta-analyses conclude that light therapy is yet to be established as a treatment to modify sleep and motor behaviour in PD.^5^^,^^6^ These meta-analyses identified only five randomised trials^5^^,^^6^ and one stated that RCTs must be conducted that follow standardised light protocols.^6^

Over the past two decades, light intervention studies in PD have implemented non-biologically-directed lights with various clinical designs.^7^, ^8^, ^9^, ^10^, ^11^, ^12^ Those studies used lights with different retinal photoreceptor activation ratios (i.e., not matching those ratios that occur in full sunlight), colour appearances (white, blue, orange, red) and intensities (ranging between 100 and 10,000 lux)^5^^,^^6^ that prohibit double-blinding. Furthermore, these light therapy interventions have been delivered at different times of the day^7^^,^^9^ and without monitoring environmental light exposure,^7^ which has all contributed to the inconclusive outcomes reported to date.^5^^,^^6^ Most importantly, none of the light interventions previously evaluated have utilised a controlled daylight condition that activates all photoreceptor classes (rods, cones and melanopsin) in the correct excitation ratios as they occur in natural daylight^13^; which is critical where light therapy is aiming to mimic the biological effects of sunlight. Daylight is important for healthy living^14^ and all photoreceptor classes are required to mediate the full effects of sleep.^15^ It is the light dependent activation of melanopsin cells, in addition to rods and cones, that encodes the huge range of ambient illuminations encountered across the 24 h day–night cycle to synchronise the biological rhythm.^16^ Moreover, emerging evidence points to a potential negative effect of artificial light on dopaminergic neurons that does not match daylight.^17^

To be most effective any light therapy must utilise spectra that target the pathological sites within the retina and circadian pathways.^18^ Evidence-based anatomical and functional studies demonstrate that photosensitive melanopsin retinal ganglion cells in the eye undergo degeneration in PD, thereby contributing to circadian disruption and clinical symptoms.^19^^,^^20^ Importantly, melanopsin cells together with rods and cone photoreceptors are responsible for relaying the ambient light information to the central clock via the retinohypothalamic tract to synchronise the circadian rhythm and modulate the sleep–wake cycle.^21^^,^^22^ Thus a significant unmet need is the application of light therapy with spectra that target the melanopsin pathology. Taken together, there is no consensus on what light therapy should be applied in PD, when it should be delivered and how or why it may work.

In this study, we used two biologically-directed lights that differentially stimulate melanopsin cells via the retinohypothalamic tract. One treatment arm matched the melanopsin excitation that would be expected from standard daylight (Day Mel), whilst the other treatment arm enhanced melanopsin excitation (Enhanced Mel) to evaluate the mechanism underpinning any clinical improvements.^23^ Importantly, both lights appeared visually similar in colour and photometric luminance to minimise the adverse effects of preference.^24^ This also allowed for the double-blinding, thus overcoming a limitation of prior light intervention studies.^5^^,^^6^ In line with the need for a precision, mechanism-based treatment approach, we also confirmed the presence of melanopsin dysfunction at baseline. Light therapy has been administered based on the rationale of circadian phase alterations in PD^8^ and based on insomnia type.^11^ It is known that the circadian system is highly susceptible to the timing of a light stimulus^25^ and recent evidence suggests that light therapy based on chronotype improves sleep.^26^ Thus, this trial aimed to optimise the timing of light intervention based on the individual patient's chronotype.

Here we report the outcomes of a phase 2, randomised, active-controlled clinical trial assessing the effect of a daylight matched and an enhanced melanopsin light therapy on sleep, wellbeing, motor and non-motor functions in PD. We aimed to determine whether a controlled daylight condition was effective in improving these clinical parameters, and whether a melanopsin boosted intervention provided any additional benefits. We evaluated the mechanism of light therapy and its direct effects via the retinohypothalamic pathway by assessing the change in sleep stages measured with polysomnography, the change in endocrine, circadian phase through melatonin onset as well as the change in the melanopsin mediated pupil light reflex.

## Methods

### Study design and participants

This TGA-regulated single centre, randomised, double-blind, parallel group, active-controlled, clinical trial was conducted at Queensland University of Technology (QUT) between February 2021 and December 2022. Participants with PD received the light intervention over 4-weeks followed by 2-weeks of no light therapy to assess its effect on primary and secondary outcomes. The study protocol was approved by the Human Research Ethics Committee at QUT (approval no: 2000000435) and adhered to the tenets of Declaration of Helsinki and Good Clinical Practice (GCP) guidelines. There were no violations to the protocol; minor deviations related to personal preferences (e.g., actigraphy swap in week 2 was done via mail and not through a personal home visit) or due to appointment scheduling changes due to either mandatory Covid-19 lock down or personal reason (e.g., sickness) in 9 occasions each. Written informed consent was provided by all participants at their baseline visit. The study followed the Consolidated Standards of Reporting Trials (CONSORT) reporting guidelines.

People with PD were eligible if they could walk unaided, were able to follow the study protocol and had no eye disease that affected melanopsin function other than PD (e.g., glaucoma, macular degeneration or diabetic retinopathy).^27^ They were excluded if they had recently travelled across two or more time zones within 90 days or took supplemental melatonin. Patients with deep brain stimulation, a medical condition other than PD that significantly affected their health, severe cognitive impairment (Addenbrooke's Cognitive examination ≤82), a recent or recurring history of musculoskeletal injury or surgery were excluded. Based on the polysomnography (PSG) assessment at baseline, people with PD were excluded if they had periodic limb movement disorder (total PLMS Arousal Index >15/h) or significant sleep apnoea (Apnea-Hypopnea Index, AHI >15 event/h that was not positional). Parkinson's severity and stage was assessed by the MDS-UPDRS and Hoehn and Yahr scores at baseline.

### Randomisation and masking

An independent data analyst who was not involved in patient testing performed the 1:1 randomisation to either Day Mel or Enhanced Mel light therapy intervention using a binary random number generator in MATLAB (MathWorks, version R2018b). The masked assignment codes were uploaded in a folder with restricted access according to the clinical trial's Data Management Plan. Participants, study sponsors, funders, investigators and staff were blinded to the type of light intervention allocation during the trial and the 8 month period of data analysis. Identical light box devices were distributed to the participants.^13^ Participants were not informed which light intervention they received at any stage during or after the trial. Data analysis was performed by an independent biostatistician (Research Methods Group, Faculty of Health, QUT) who was also masked to the interventions. The light intervention allocation was made available to investigators and staff after the data were analysed.

### Procedures

The concept of photoreceptor-directed light therapy has been recently described.^13^ In short, the technology was incorporated in a portable wide-field lighting device developed for this clinical trial and comprises multiple limited-bandwidth [peak wavelength (full width at half maximum): 448 nm (17 nm); 469 nm (23 nm); 506 nm (28 nm); 520 nm (30 nm); 598 nm (15 nm) and 654 nm (18 nm)] primary lights that can generate different combinations of photoreceptor-directed light to selectively alter the melanopsin excitation between a controlled daylight (Day Mel) and an enhanced melanopsin state (Enhanced Mel). Spectral, photometric and radiometric calibrations of the lighting devices were completed for a time equivalent to the duration of the light supplementation protocol using a spectroradiometer (StellarNet, Tampa, FL, USA), ILT1700 Research Radiometer (International Light Technologies, Inc., Peabody, MA, USA) and SpectraScan PR655 (Jadak, Syracuse, NY) as per recommended procedures.^28^ All primary lights were specified with reference to the excitations of the five photoreceptor classes using the CIE S026/E: 2018 standardised L-, M- and S-cone, rhodopsin (R) and melanopsin (i) spectral sensitivity functions (SMLRi). The spectral distributions of natural daylights as defined by the Illuminating Engineering Society (IES) TM-30 standard were then correlated with the photoreceptor spectral sensitivity functions to give five SMLRi values for each natural daylight spectrum. The SMLRi photoreceptor excitations of the primaries for the corresponding Day Mel and Enhanced Mel conditions were computed, verified, and the protocol written in Arduino script (Arduino IDE 1.8.10) which contained the calibrated pulse-width modulation values to control the primary light outputs, and the protocol timing parameters. The visual appearance of both lights closely matches a correlated colour temperature of 3500 K. The Day Mel condition matches the biological effects of all photoreceptor excitations (melanopsin, rods and cones) in the human eye as they occur in 3500 K sunlight to provide a natural daylight control. The Enhanced Mel causes a circadian equivalent increase in melanopsin excitation of 5500 K (Δ24% melanopsin Weber contrast between the two states).^13^

Correct use of the device use was demonstrated to participants (30 cm viewing distance) and written instructions were provided. The timed light exposure was started with a button press and the device automatically turned off after 30 min of light delivery. It was powered by a low voltage power pack (up to 24VDC 4 A or equivalent) that complied with Australian national safety standards. The output light levels (6750 cd m^−2^; 16,500 lux) measured at the position of the cornea of the eye at a 30 cm viewing distance were safe and lower than levels commonly encountered outside on a sunny day (32,000–100,000 lux). Standard optical corrections ensured that there was no risk of eye damage from ultra-violet light (i.e., no blue light hazard). Each device was calibrated and tested for electrical or optical faults by a Research Engineer who was not involved in data collection. For adherence to the light intervention, participants were alerted by an auditory signal from the light box to lift their arm wearing the actigraph to the front of the light to register their compliance at the start, halfway through the exposure (15 min) and 2 min before the end of 30 min light exposure.

Detailed procedures and methods are available in the Appendix (pp 4–10). Briefly, participants completed three laboratory visits at QUT: a baseline visit, at week 4 (following intervention) and at week 6 (2 weeks after discontinuing the intervention). In addition, participants were visited at home by staff on four occasions (weeks 1 and 2 for equipment and compliance checks and at weeks 4 and 6 to set up the overnight polysomnography). During the baseline visit, participants completed the Addenbrooke's cognitive examination and underwent an eye assessment including retinal nerve fibre layer (RNFL) and retinal (macula) thickness scans (RS3000-Advance, HD OCT, Nidek, USA). In addition, each individual completed the Morningness Eveningness Questionnaire to determine their chronotype and guide the recommended timing of the light intervention. At all time points (baseline, weeks 4 and 6), the following tests were performed: pupillography to verify melanopsin function/deficits (post-illumination pupil response, PIPR), in–home polysomnography (Somte PSG, Compumedics Limited, Australia) and salivary dim light melatonin onset (DLMO) on the night of the PSG. Validated questionnaires assessed sleep, affect, quality of life: the Pittsburgh Sleep Quality Index (PSQI), Epworth Sleepiness Scale (ESS), Parkinson's Disease Sleepiness Scale (PDSS), Beck Depression Inventory-II (BDI-II), Parkinson's Diseases Questionnaire (PDQ-39) and the Movement Disorder Society-Unified Parkinson's Disease Rating Scale (MDS-UPDRS). Data on gait (ZenoMetrics, LLC, Peekskill, New York), balance (Vicon Motion Systems, Centennial, CO) and tremor (resting and postural) recorded by inertial measurement units (IMUs) attached to the index fingers of both hands (Vicon, Oxford Metrics, UK) were also collected.

Based on the chronotype of the participants, the light intervention was recommended between 5 am and 7 am as per MEQ guidelines (Appendix, p6). Subjective, self-reported daily data indicated that participants implemented the light therapy on average (SD) at 7:11 am (±1:35 h), [Day Mel on average at 7:26 am (±1:37 h) and Enhanced Mel on average at 6:56 am (±1:33 h)] (Table S2), and over 26.04 (±3.61) days. Participants wore an actigraph (GENE Active V3.1, Activinsights Ltd, UK) on their non-dominant wrist to capture ambient light exposure over the 6-week study duration. Both light invention groups had similar environmental light exposure as measured with actigraphy over the 6 week study duration [F (5, 255) = 1.89, p = 0.096] (Fig. S1) indicating that the outcomes were not due to between group differences in ambient light exposure. Illumination levels measured by actigraphy also served as a control for dim light melatonin collection and confirmed that the saliva was collected in dim light and on average (mean (SEM)) at 28.53 (2.29) lux.

We confirmed melanopsin dysfunction as determined by the 6s post-illumination pupil response (PIPR), in both groups at baseline compared to healthy age-matched control data [(F (1, 74) = 10.26, p = 0.0001), post-hoc PIPR% mean (95% CI), Day Mel: 14.07 (5.68–22.45), p = 0.0003; Enhanced Mel: 13.35 (5.03–21.68), p = 0.001)] (Fig. S2A). At week 4 and 6, there was an improvement in melanopsin function (up to 5%)^19^ in the Enhanced Mel group (Fig. S2).

There were no safety concerns or severe adverse events related to the light interventions. One participant reported a mild headache during the first 10 min of light exposure over the 4 weeks of light intervention, and another participant experienced sleep problems for one night.

### Outcomes

All outcome measures were compared between the following time points and between groups: baseline vs week 4 and baseline vs week 6. The primary endpoints were a change in the means of any of the polysomnography parameters (sleep stages % N1, N2, N3, REM, total sleep time, sleep onset latency, sleep efficiency, time awake, REM latency, total PLMS arousal index), and a change in the mean of the salivary Dim light Melatonin Onset (DLMO decimal time). Key secondary endpoints included a change of the mean sleep questionnaire scores (PSQI, ESS, PDSS). Other secondary endpoints included a change in mean motor function (gait, balance, resting and postural tremor). Exploratory endpoints were a change in mean affect (Beck Depression Inventory-II), quality of life (PDQ-39) and MDS-UPDRS (total and motor-section III) scores (Appendix, pp4–7). Safety and adverse events were assessed at each laboratory and home visit.

### Statistical analysis

We enrolled 60 participants based on power and sample size calculations^29^ of the mean difference data (effect size) that achieved a clinically relevant effect on the circadian system from prior studies in PD and a healthy cohort.^7^^,^^9^^,^^19^ The final sample size was determined based on the primary endpoints needing the largest sample size [DLMO: mean difference 1.2 h^19^ (±0.65 h)], and which required a minimum sample of 28 persons per group. This included adjustments for the longitudinal nature of the data using an inflation factor^29^ of 3.8 in order to detect a significant difference assuming a type I error of 5% (two tailed) and a type II error of 10% (90% power).

**Fig. 1:**
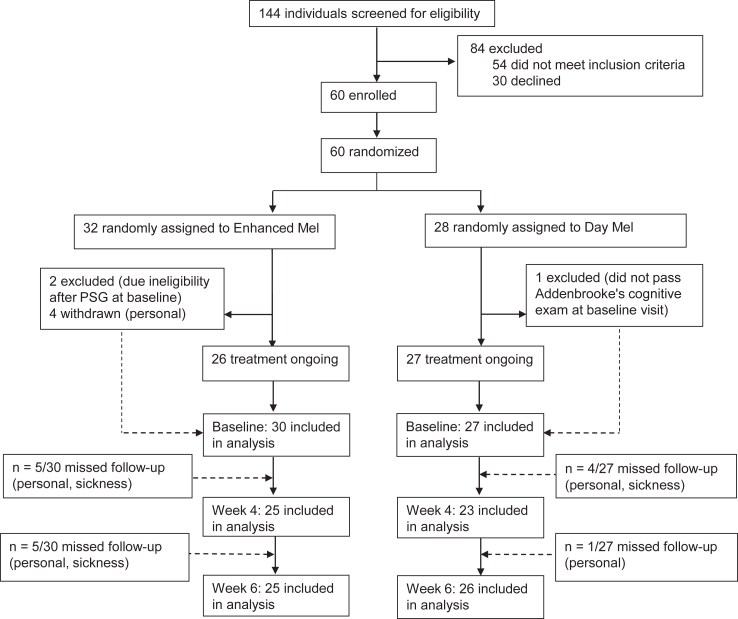
**Trial profile**. Analysis and follow up profile for primary outcome (polysomnography).

Data analysis was undertaken between January 2023 and July 2023. Descriptive statistics were calculated for demographic characteristics of the study cohort and by study group. The data were analyzed by fitting a linear mixed model. Mixed models were used to account for the longitudinal nature of the data, using intervention group, time point and interactions as explanatory variables to the primary, secondary and exploratory outcome measures in R applying packages as described in the Appendix (p5). Furthermore, the fitted mixed models were used in *post hoc* analyses with respect to group and time point to explore potential differences due to these two variables and their interaction. The mixed models were adjusted for baseline characteristics including age, sex, Hoehn and Yahr disease stage, PD duration and levodopa equivalent daily dosage (LEDD)^7^ based on recommended reporting standards of RCTs^24^ and CONSORT guidelines. The number of participants analysed for each outcome measure are provided in Fig. 1 (PSG), Tables 2 and 3 and Table S1. There was a small amount of missing data across the outcome measures due to government mandated Covid-19 restrictions, and for personal reasons of the patients. To account for missing data, the mixed model used all available data and information in the analyses, using (Restricted) Maximum Likelihood as the optimization procedure.^30^ Furthermore, we analysed the available data with covariate adjustment for all outcome variables.^31^ The overall effect was determined by the mean difference between the groups at each timepoint and the change at week 4 and week 6 for all patients in both randomised groups. The mean differences (95% CI) for the main effects are reported over all follow up unless otherwise specified; the group comparisons were Enhanced Mel vs Day Mel as a reference and the time comparison were week 4 and week 6 with baseline as the reference. To document environmental light exposure, averaged weekly actigraphy lux data were compared across the 6-weeks of the study period (Appendix, p5). Baseline melanopsin function (PIPR) in people with PD was compared to a normative age-matched data set from our laboratory. All statistical tests were done using a two tailed 5% significance level. Trial auditing and monitoring was provided by QUT's Clinical Trial Unit who was independent from the investigators and clinical staff. The trial was registered with ACTRN (no: ACTRN12621000077864).

### Role of the funding source

The funder of the study had no role in study design, data collection, data analysis, data interpretation, or writing of the report.

## Results

Participants were recruited between February 4th 2021 and August 8th 2022 (Fig. 1). Out of 144 consecutively screened people with PD from Parkinson's support groups in Brisbane and South-East Queensland, Australia, 30 individuals declined, 54 were ineligible and 60 were enrolled and randomised to either Day Mel (n = 28) or Enhanced Mel (n = 32). The baseline characteristics of the participants are outlined in Table 1. Of the 60 randomised participants, three were excluded at baseline (one participant failed due to their performance on the Addenbrooke's cognitive examination and two participants did not meet the baseline PSG inclusion criteria due to significant sleep apnea). During the light intervention, four participants withdrew due to personal reasons after 1–3 weeks of light therapy (Fig. 1).

**Table 1:** Baseline demographics and disease characteristics.

	All: n = 57	Day Mel: n = 27	Enhanced Mel: n = 30
Age, years; Median (Q1–Q3)	68 (62–75)	72 (65–75)	66 (62–74)
Age, years; Mean (SD)	68.1 (7.9)	69.4 (7.7)	66.8 (7.9)
Sex: Female	24 (42.1%)	10 (37.0%)	14 (46.7%)
Sex: Male	33 (57.9%)	17 (63.0%)	16 (53.3%)
H&Y stage 1	23 (40.4%)	12 (37.0%)	13 (43.4%)
H&Y stage 2	32 (56.1%)	16 (59.3%)	16 (53.3%)
H&Y stage 3	2 (3.5%)	1 (3.7%)	1 (3.3%)
Disease duration, years; Median (Q1–Q3)	4 (2–7)	4.0 (2–5.5)	4.5 (1–7.8)
Levodopa daily dosage; Median (Q1–Q3)	500 (250–750)	564 (362.5–800)	475 (205.0–730)
Retinal macula thickness, left eye; Median (Q1–Q3)	279 (256–301)	270 (258.0–289.0)	286 (255.2–305.5)
Retinal macula thickness, left eye; Mean (SD)	284.8 (41.4)	280.4 (30.4)	288.9 (49.4)
Retinal nerve fibre thickness, left eye; Median (Q1–Q3)	95 (88–101)	94 (87.0–99.5)	95 (88.2–102.0)
Retinal nerve fibre thickness, left eye; Mean (SD)	96 (15.8)	96.2 (20.5)	95.7 (10.2)
Chronotype: moderate morning	28 (49.1%)	14 (51.9%)	14 (46.7%)
Chronotype: intermediate	22 (38.6%)	12 (44.4%)	10 (33.3%)
Chronotype: definitive morning	6 (10.5%)	0 (0.0%)	6 (20.0%)
Chronotype: moderate evening	1 (1.8%)	1 (3.7%)	0 (0.0%)

Categorical variables are summarised as frequency (percentage). Continuous variables are summarised by quantiles (Median, Q1–Q3) and means with standard deviations. Day Mel, Daylight Melanopsin group. Enhanced Mel, Enhanced Melanopsin group.

The mean and 95% CI at each time point (BL, week 4 and week 6) with main effects (group and time) and interactions (group by time) with p values are shown in Tables 2 and 3 and Table S1. There was no significant difference in any outcome measure between randomised groups overall or at any individual timepoint during follow-up. For the co-primary outcomes, the mean (95% CI) for PSG, N3% was 24.15 (19.82–28.48) for Day Mel (n = 23) and 19.34 (15.20–23.47) for the Enhanced Mel group (n = 25) in week 4 (p = 0.12); and 21.13 (16.99–25.28) for Day Mel (n = 26) and 18.48 (14.34–22.62) for the Enhanced Mel group (n = 25) in week 6, (p = 0.37). The mean (95% CI) DLMO (decimal time) was 19.82 (19.20–20.44) for Day Mel (n = 22) and 19.44 (18.85–20.04) for the Enhanced Mel group (n = 24) in week 4 (p = 0.38); and 19.90 (19.27–20.53) for Day Mel (n = 23) and 19.04 (18.44–19.64) for the Enhanced Mel group (n = 25) in week 6 (p = 0.05). However, there were significant changes in primary, secondary and exploratory outcomes for both randomised groups combined at specific timepoints, although in many cases, tests of interaction between treatment and time were not significant.

**Fig. 2:**
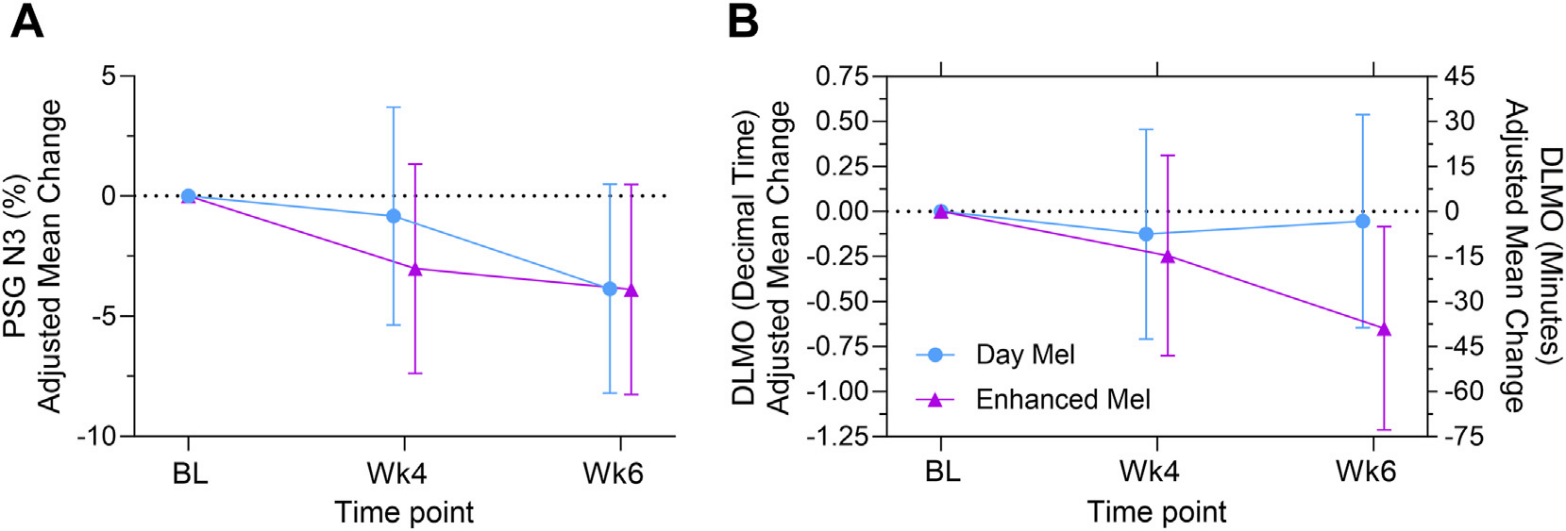
**Effect of light therapy on primary endpoints**. Model-predicted adjusted mean (95% CI) change from baseline for stage N3 sleep recorded by polysomnography (A) and dim light melatonin onset (B). Error bars represent 95% CIs. Day Mel, Daylight Melanopsin group (blue circles with blue error bars). Enhanced Mel, Enhanced Melanopsin group (purple triangles with purple error bars). PSG, Polysomnography. DLMO, Dim Light Melatonin Onset. BL, Baseline. Wk4, Week 4. Wk6, Week 6.

In both groups, the primary outcome measure PSG showed a change in week 6, with a significant improvement of the mean restorative, deep sleep stage component (N3) [model-predicted adjusted mean difference to baseline (95% CI): −3.87 (−6.91 to −0.83), p = 0.04] (Table 2). There was a trend for greater improvement in N3 in the Enhanced Mel group compared to the Day Mel in week 4 (Fig. 2A). The remaining PSG outcomes were not significantly affected by intervention type or time.

**Table 2:** Primary outcome measures, estimated by mixed models.

Time point	Day Mel	Enhanced Mel	Mean difference-group (Enhanced Mel—Day Mel)	p value group	Time point	Mean difference-time	p value time	p value group x Time
**Primary outcomes**								
**Polysomnography**								
Total sleep time (min)								
BL	310.84 (281.67–340.00); 27	302.65 (275.08–330.22); 30	−8.19 (−48.10 to 31.73)	0.69	WK4-BL	−5.40 (−29.43 to 18.63)	0.90	0.90
WK4	305.82 (274.73–336.91); 23	296.86 (267.18–326.54); 25	−8.96 (−51.74 to 33.83)	0.68	WK6-BL	−13.95 (−37.51 to 9.61)	0.48	
WK6	292.28 (262.73–321.84); 26	293.31 (263.57–323.05); 25	1.03 (−40.56 to 42.61)	0.96	WK6–Wk4	−8.54 (−33.10 to 16.02)	0.78	
Sleep onset latency (min)								
BL	22.23 (12.77–31.69); 27	28.15 (19.34–36.97); 30	5.93 (−6.94 to 18.79)	0.37	WK4-BL	−3.59 (−11.82 to 4.64)	0.67	0.42
WK4	17.66 (7.77–27.67); 23	25.54 (16.00–35.08); 25	7.88 (−5.89 to 21.64)	0.26	WK6-BL	−0.92 (−9.03 to 7.19)	0.97	
WK6	25.63 (16.16–35.09); 26	22.91 (13.35–32.48); 25	−2.72 (−16.06 to 10.63)	0.69	WK6–Wk4	2.67 (−5.74 to 11.08)	0.81	
Stage REM latency (min)								
BL	109.63 (77.64–141.62); 27	113.77 (83.56–143.99); 30	4.14 (−39.64 to 47.92)	0.85	WK4-BL	5.11 (−24.45 to 34.67)	0.94	0.96
WK4	110.89 (75.71–146.08); 23	122.73 (89.91–155.54); 25	11.83 (−36.05 to 59.72)	0.63	WK6-BL	−8.38 (−20.41 to 37.17)	0.84	
WK6	100.82 (68.34–133.29); 26	105.82 (72.92–138.73); 25	5.01 (−40.85 to 50.86)	0.83	WK6–Wk4	−13.49 (−43.73 to 16.75)	0.66	
Sleep efficiency (%)								
BL	71.10 (65.66–76.54); 27	70.33 (65.19–75.47); 30	−0.77 (−8.21 to 6.67)	0.84	WK4-BL	−2.49 (−6.53 to 1.55)	0.45	0.25
WK4	71.59 (65.85–77.33); 23	64.85 (59.37–70.33); 25	−6.74 (−14.64 to 1.16)	0.10	WK6-BL	−1.10 (−2.86 to 5.06)	0.85	
WK6	69.88 (64.38–75.38); 26	69.35 (63.86–74.84); 25	−0.53 (−8.23 to 7.18)	0.89	WK6–Wk4	1.39 (−2.73 to 5.51)	0.79	
Time awake (min)								
BL	106.29 (81.63–130.94); 27	103.92 (80.62–127.22); 30	−2.37 (−36.07 to 31.34)	0.89	WK4-BL	12.49 (−30.19 to 5.21)	0.35	0.13
WK4	101.58 (75.63–127.53); 23	133.61 (108.84–158.38); 25	32.03 (−3.65 to 67.70)	0.08	WK6-BL	0.27 (−17.08 to 17.62)	1.00	
WK6	104.83 (79.91–129.75); 26	105.92 (81.12–130.72); 25	1.09 (−33.77 to 35.94)	0.95	WK6–Wk4	−12.22 (−30.29 to 5.85)	0.39	
Stage N1 sleep (%)								
BL	8.68 (6.60–10.76); 27	11.05 (9.08–13.01); 30	2.37 (−0.48 to 5.21)	0.11	WK4-BL	−0.74 (−0.87 to 2.35)	0.64	0.07
WK4	8.58 (6.38–10.78); 23	9.67 (7.57–11.77); 25	1.09 (−1.94 to 4.12)	0.48	WK6-BL	0.85 (−0.72 to 2.42)	0.54	
WK6	11.38 (9.27–13.48); 26	10.05 (7.94–12.16); 25	−1.33 (−4.28 to 1.63)	0.38	WK6–Wk4	1.59 (−0.04 to 3.22)	0.14	
Stage N2 sleep (%)								
BL	49.35 (45.02–53.68); 27	48.56 (44.47–52.65); 30	−0.79 (−6.71 to 5.13)	0.79	WK4-BL	2.64 (−0.44 to 5.72)	0.22	0.14
WK4	50.29 (45.73–54.84); 23	52.90 (48.55 to 57.25); 25	2.61 (−3.65 to 8.87)	0.42	WK6-BL	2.60 (−0.42 to 5.62)	0.21	
WK6	48.91 (44.54–53.29); 26	54.21 (49.85–58.56); 25	5.29 (−0.82 to 11.41)	0.09	WK6–Wk4	−0.03 (−3.17 to 3.11)	1.00	
Stage N3 sleep (%)								
BL	24.99 (20.89–29.08); 27	22.36 (18.49–26.23); 30	−2.63 (−8.23 to 2.97)	0.36	WK4-BL	−1.93 (−5.05 to 1.19)	0.45	0.74
WK4	24.15 (19.82–28.48); 23	19.34 (15.20–23.47); 25	−4.82 (−10.77 to 1.14)	0.12	WK6-BL	−3.87 (−6.91 to −0.83)	**0.04**	
WK6	21.13 (16.99–25.28); 26	18.48 (14.34–22.62); 25	−2.66 (−8.46 to 3.15)	0.37	WK6–Wk4	−1.94 (−5.12 to 1.24)	0.46	
REM (%)								
BL	16.99 (13.58–20.41); 27	18.03 (14.80–21.25); 30	1.03 (−3.63 to 5.69)	0.67	WK4-BL	0.04 (−2.33 to 2.41)	1.00	0.53
WK4	17.00 (13.42–20.58); 23	18.11 (14.69–21.53); 25	1.11 (−3.81 to 6.03)	0.66	WK6-BL	0.42 (−1.89 to 2.73)	0.93	
WK6	18.57 (15.12–22.02); 26	17.28 (13.86 –20.70); 25	−1.29 (−6.10 to 3.52)	0.60	WK6–Wk4	0.37 (−2.04 to 2.78)	0.95	
Total arousal index (/hour)								
BL	2.78 (1.07–4.49); 27	1.43 (−0.19 to 3.04); 30	−1.36 (−3.69 to 0.98)	0.26	WK4-BL	−0.78 (−1.96 to 0.40)	0.40	0.54
WK4	1.49 (−0.31 to 3.28); 23	1.17 (−0.54 to 2.88); 25	−0.32 (−2.78 to 2.15)	0.80	WK6-BL	−0.47 (−1.63 to 0.69)	0.70	
WK6	1.70 (−0.03 to 3.43); 26	1.56 (−0.16 to 3.27); 25	−0.14 (−2.55 to 2.27)	0.91	WK6–Wk4	0.30 (−0.90 to 1.50)	0.87	
**DLMO (decimal time)**								
BL	19.95 (19.33–20.57); 24	19.69 (19.12–20.26); 27	−0.26 (−1.10 to 0.57)	0.54	WK4-BL	−0.19 (−0.58 to 0.20)	0.63	0.32
Wk4	19.82 (19.20–20.44); 22	19.44 (18.85–20.04); 24	−0.38 (−1.23 to 0.47)	0.38	WK6-BL	−0.35 (−0.76 to 0.06)	0.21	
Wk6	19.90 (19.27–20.53); 23	19.04 (18.44–19.64); 25	−0.86 (−1.72 to 0.01)	0.05	WK6–Wk4	−0.17 (−0.56 to 0.22)	0.70	

Data are average means (95% CIs) and the number of participants for all the primary outcome measures. BL, Baseline. Wk4, Week 4. Wk6, Week 6. REM, Rapid Eye Movement. DLMO, Dim Light Melatonin Onset. Day Mel, Daylight Melanopsin group. Enhanced Mel, Enhanced Melanopsin group. Bold p values indicate statistically significant results.

The co-primary outcome, dim light melatonin onset (DLMO) was phase-advanced in both groups (Table 2). There was a trend towards a greater phase advance on average in the Enhanced Mel compared to the Day Mel group in week 4 (15 min vs 8 min, respectively), which became longer in week 6 (39 min vs 3 min, respectively) (Fig. 2B). When considering the model-predicted adjusted mean (95% CI) change from baseline in the Enhanced Mel group, the DLMO was significantly phase advanced in week 6 [−0.65 (−1.21 to −0.09), p = 0.02].

**Fig. 3:**
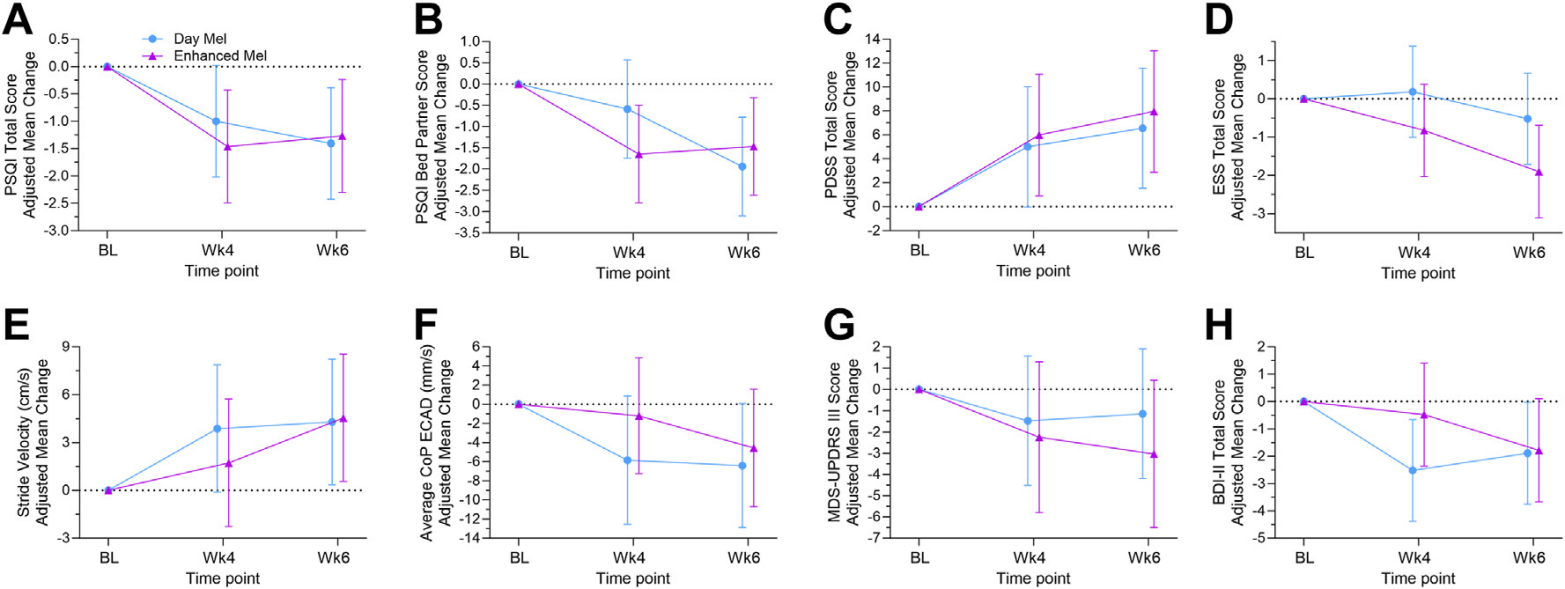
**Effect of light therapy on secondary and exploratory endpoints**. Model-predicted adjusted mean (95% CI) change from baseline for PSQI total score (A), participant's sleep quality reported by their bed partner (B), PDSS total score (C) and ESS total score (D), stride velocity (E), balance average centre of pressure (CoP) standing on foam surface with eyes closed and arms down (ECAD) (F), MDS-UPDRS motor score (G) and BDI-II total score (H). Error bars represent 95% CIs. Day Mel, Daylight Melanopsin group (blue circles with blue error bars). Enhanced Mel, Enhanced Melanopsin group (purple triangles with purple error bars). PSQI, Pittsburgh Sleep Quality Index. PDSS, Parkinson's Disease Sleep Scale. ESS, Epworth Sleepiness Scale. MDS-UPDRS, Movement Disorder Society-Unified Parkinson's Disease Rating Scale. BDI, Beck Depression Inventory. BL, Baseline. Wk4, Week 4. Wk6, Week 6.

For the key secondary endpoints (PSQI, ESS, PDSS) (Table 3), significant mean differences to baseline were found at week 4 and week 6 in both intervention groups [model-predicted adjusted mean difference to baseline (95% CI)]: the PSQI total score significantly improved (lower scores) [week 4: −1.23 (−1.96 to −0.50), p = 0.003, week 6: −1.34 (−2.07 to −0.61), p = 0.001]; also the PSQI bedpartner sub-score representing the partner's perception of the patient's sleep quality was significantly lower reflecting this improvement [week 4: −1.12 (−1.92 to −0.32), p = 0.02, week 6: −1.71 (−2.51 to −0.91), p < 0.0001]. The PDSS total score significantly improved (higher values) in both groups at both time points [week 4: 5.50 (1.97–9.03), p = 0.01, week 6: 7.26 (3.73–10.79), p < 0.0001]. Daytime sleepiness as assessed by ESS score demonstrated significantly lower (better) scores [week 6: −1.21 (−2.05 to −0.37), p = 0.02]. The model-predicted adjusted mean (95% CI) change from baseline for each group is shown in Fig. 3A–D.

**Table 3:** Key secondary and secondary outcome measures, estimated by mixed models.

Time point	Day Mel	Enhanced Mel	Mean difference-group (Enhanced Mel—Day Mel)	p value group	Time point	Mean difference-time	p value time	p value group x time
**Key secondary outcomes**								
PSQI-Global score								
BL	8.52 (7.14–9.90); 27	8.68 (7.37–9.99); 30	0.16 (−1.73 to 2.04)	0.87	WK4-BL	−1.23 (−1.96 to −0.50)	**0.003**	0.70
WK4	7.52 (6.14–8.90); 27	7.22 (5.87–8.57); 26	−0.31 (−2.21 to 1.60)	0.76	WK6-BL	−1.34 (−2.07 to −0.61)	**0.001**	
WK6	7.11 (5.73–8.49); 27	7.41 (6.06–8.76); 26	0.30 (−1.61 to 2.20)	0.76	WK6–Wk4	−0.11 (−0.84 to 0.62)	0.95	
PSQI-Bed partner sleep quality								
BL	4.98 (3.58–6.39); 18	4.07 (2.74–5.40); 19	−0.91 (−2.78 to 0.96)	0.34	WK4-BL	−1.12 (−1.92 to −0.32)	**0.02**	0.17
WK4	4.39 (2.99–5.80); 17	2.42 (1.06–3.79); 17	−1.97 (−3.86 to −0.08)	0.05	WK6-BL	−1.71 (−2.51 to −0.91)	**<0.0001**	
WK6	3.04 (1.64–4.44); 17	2.60 (1.23–3.96); 17	−0.44 (1.45 to 0.65)	0.65	WK6–Wk4	−0.59 (−1.39 to 0.21)	0.33	
ESS total score								
BL	8.25 (6.33–10.17); 27	9.48 (7.66–11.30); 30	1.23 (−1.39 to 3.84)	0.36	WK4-BL	−0.32 (−1.16 to 0.52)	0.74	0.25
WK4	8.44 (6.52–10.36); 27	8.65 (6.79–10.52); 26	0.22 (−2.43 to 2.86)	0.87	WK6-BL	−1.21 (−2.05 to −0.37)	**0.02**	
WK6	7.73 (5.81–9.66); 27	7.58 (5.71–9.44); 26	−0.16 (−2.80 to 2.49)	0.91	WK6–Wk4	−0.89 (−1.73 to −0.05)	0.10	
PDSS total score								
BL	99.85 (91.68–108.03); 27	99.18 (91.43–106.93); 30	−0.68 (−11.82 to 10.47)	0.91	WK4-BL	5.50 (1.97–9.03)	**0.01**	0.92
WK4	104.86 (96.68–113.03); 27	105.17 (97.25–113.09); 26	0.31 (−10.94 to 11.57)	0.96	WK6-BL	7.26 (3.73–10.79)	**<0.0001**	
WK6	106.41 (98.23–114.58); 27	107.14 (99.22–115.06); 26	0.73 (−10.52 to 11.99)	0.90	WK6–Wk4	1.76 (−1.79–5.31)	0.59	
**Other secondary outcomes**								
**GAIT**								
Stride velocity (cm/s)								
BL	106.96 (99.57–114.35); 27	113.66 (106.68–120.65); 30	6.71 (−3.34 to 16.75)	0.20	WK4-BL	2.80 (0.02–5.58)	0.13	0.66
Wk4	110.83 (103.38–118.28); 25	115.39 (108.26–122.51); 26	4.56 (−5.62 to 14.74)	0.38	WK6-BL	4.42 (1.66–7.18)	**0.01**	
Wk6	111.25 (103.83–118.67); 26	118.20 (111.08–125.33); 25	6.96 (−3.20 to 17.12)	0.18	WK6–Wk4	1.61 (−1.21 to 4.43)	0.51	
Total double support (%)								
BL	24.66 (23.27–26.05); 27	24.01 (22.69–25.32); 30	−0.65 (−2.54 to 1.24)	0.50	WK4-BL	−0.14 (−0.59 to 0.31)	0.81	0.70
Wk4	24.53 (23.13–25.93); 25	23.85 (22.52–25.19); 26	−0.68 (−2.59 to 1.23)	0.49	WK6-BL	−0.59 (−1.04 to −0.14)	**0.03**	
Wk6	24.25 (22.85–25.64); 26	23.24 (21.90–24.57); 25	−1.01 (−2.91 to 0.90)	0.30	WK6–Wk4	−0.45 (−0.92 to 0.02)	0.15	
**Balance**								
Average CoP (mm/s)								
Stand foam (EOAD)								
BL	43.22 (39.95–46.49); 26	43.76 (40.68–46.84); 30	0.54 (−3.89 to 4.98)	0.81	WK4-BL	−0.45 (−1.72 to 0.82)	0.77	0.89
Wk4	43.03 (39.76–46.30); 26	43.04 (39.90–46.19); 25	0.01 (−4.47 to 4.50)	1.00	WK6-BL	0.73 (−0.56 to 2.02)	0.51	
Wk6	43.92 (40.65–47.19); 25	44.51 (41.35–47.68); 24	0.59 (−3.90 to 5.09)	0.80	WK6–Wk4	1.18 (−0.11 to 2.47)	0.18	
Stand foam (ECAD)								
BL	81.63 (70.08–93.19); 23	68.76 (58.30–79.21); 30	−12.88 (−28.22 to 2.46)	0.11	WK4-BL	−3.52 (−7.97 to 0.93)	0.27	0.59
Wk4	75.79 (64.18–87.41); 22	67.55 (56.88–78.22); 25	−8.25 (−23.78 to 7.29)	0.30	WK6-BL	−5.48 (−9.89 to −1.07)	**0.04**	
Wk6	75.23 (63.71–86.75); 24	64.19 (53.47–74.91); 24	−11.04 (−26.53 to 4.45)	0.17	WK6–Wk4	−1.96 (−6.41 to 2.49)	0.67	
**Tremor**								
Dominant frequency (Hz)-Both hands								
Sit, rest								
BL	6.19 (5.78–6.60); 26	6.11 (5.73–6.50); 30	−0.08 (−0.64 to 0.48)	0.79	WK4-BL	0.28 (0.01–0.55)	0.12	0.46
Wk4	6.33 (5.91–6.74); 26	6.53 (6.12–6.95); 25	0.21 (−0.38 to 0.79)	0.49	WK6-BL	0.26 (−0.01 to 0.53)	0.15	
Wk6	6.41 (6.01–6.82); 27	6.42 (6.00–6.83); 24	0.00 (−0.57 to 0.58)	0.99	WK6–Wk4	−0.01 (−0.28 to 0.26)	0.99	
Sit, postural								
BL	5.92 (5.57–6.27); 26	5.70 (5.38–6.03); 29	−0.21 (−0.69 to 0.26)	0.38	WK4-BL	0.04 (−0.16 to 0.24)	0.92	0.11
Wk4	5.76 (5.41–6.11); 26	5.94 (5.60–6.28); 25	0.19 (−0.30 to 0.67)	0.46	WK6-BL	0.04 (−0.16 to 0.24)	0.91	
Wk6	5.85 (5.50–6.19); 27	5.86 (5.51–6.20); 23	0.01 (−0.48 to 0.50)	0.98	WK6–Wk4	0.00 (−0.20 to 0.20)	1.00	

Data are average means (95% CIs) and the number of participants for all the secondary outcome measures. BL, Baseline. Wk4, Week 4. Wk6, Week 6. Day Mel, Daylight Melanopsin group. Enhanced Mel, Enhanced Melanopsin group. PSQI, Pittsburgh Sleep Quality Index. ESS, Epworth Sleepiness Scale. PDSS, Parkinson's Disease Sleep Scale. CoP, Center of Pressure on force plate. EOAD, Eyes Open Arms Down. ECAD, Eyes Closed Arms Down. Bold p values indicate statisitcally significant results.

Both light interventions also significantly improved the other secondary outcomes (Table 3) including [model-predicted adjusted mean difference to baseline in week 6 (95% CI): stride velocity: 4.42 (1.66–7.18), p = 0.01; total double support: −0.59 (−1.04 to −0.14), p = 0.03], and balance (average centre of pressure on foam with ECAD) [−5.48 (−9.89 to −1.07), p = 0.04]. The MDS-UPDRS motor (III) (Table S1) mean score showed a trend of improvement in the Enhanced Mel group [−6.43 (−12.85 to −1.01), p = 0.05]. The model-predicted adjusted mean (95% CI) change from baseline for each group for the motor data is shown in Fig. 3E–G. Resting and postural tremor (dominant frequency) were not significantly different with either intervention at any time point (Table 3).

The model-predicted adjusted mean difference to baseline of exploratory outcomes (the Parkinson's Disease Quality of Life Questionnaire (PDQ-39) [week 4: −5.38 (−7.95 to −2.81), p < 0.0001, week 6: −6.81 (−9.38 to −4.24), p < 0.0001], Beck Depression Inventory (BDI-II) [week 6: −1.84 (−3.15 to −0.53), p = 0.02] and MDS-UPDRS (total score) [week 4: −5.81 (−9.40 to −2.22), p = 0.01, week 6: −6.51 (−10.06 to −2.96), p = 0.002], also improved significantly in both light intervention groups (Table S1). The model-predicted adjusted mean (95% CI) change from baseline for each group for the BDI-II is shown in Fig. 3H.

## Discussion

This is the first RCT to implement a precision medicine, mechanism and chronotype guided light intervention. We demonstrate that the controlled daylight significantly improved non-motor symptoms (restorative deep sleep, affect) and wellbeing, with the booster melanopsin daylight not providing additional benefits but showing a trend for greater physiological effects than the controlled daylight on the primary endpoints. Notably, the positive effects of both light interventions were independent of the environmental light exposure, as continuously monitored with actigraphy and improvements were sustained 2 weeks after light cessation. Another novel observation was the positive perception by caregivers of the improved sleep quality, reinforcing the clinical relevance of this trial's findings. Caregivers also reported their partner had fewer episodes of confusional arousal or restlessness during the night. The beneficial effects of both interventions on affect and quality of life underpin the clinical impact of this RCT's findings on people with PD. Other outcomes included a significant improvement in motor functions (e.g. stride velocity) and MDS-UPDRS scores in both intervention groups.

This study shows that by stimulating the remaining retinal neuronal networks with daylight can potentially compensate for the melanopsin neuronal degeneration in people with PD,^19^^,^^20^ to generate positive clinical outcomes. We infer that the functional benefits of the light interventions with spectra that matched or enhanced the retinohypothalamic pathway activity in daylight, may be due to improved melanopsin signalling through projections directly or indirectly to the brain's sleep and mood centres. These include direct projections to the suprachiasmatic nucleus (SCN), as well as to sleep promoting and mood centres including the ventrolateral preoptic nucleus (VLPO), lateral habenula (LHb), ventral tegmental area (VTA), respectively, whilst indirectly acting via the SCN as a conduit for transmitting light signals to these brain areas.^32^ Importantly, that some of the improvements were sustained at week 6 or became significant at week 6, points to a physiological characteristic of melanopsin cells which are known for their long-term integration of light and signalling for several hours, even after only short burst of light.^33^ As such, the melanopsin pathway can modulate upstream effects in the brain over longer periods of time.^33^

The significant improvement of the objectively measured restorative, deep sleep stage (N3) is consistent with the positive effects of light reaching the brain's sleep regulating centres. This significant shift to normal, age-matched values^34^ after light intervention, is in line with the subjective improvement in sleep behaviour, as evaluated by the sleep questionnaires (Table 2, Fig. 3A–D). Deep sleep mostly occurs in the early sleep hours of the night and represents a restorative, recovery phase for the body, with greater metabolic waste product removal at the cellular level.^35^ This increased rest could potentially result in less dopamine depletion overnight^36^ and the improved motor function, which was also clinically meaningful^37^^,^^38^ (Fig. 3E–G, Table S1). Overall, a better deep sleep can lead to the observed improved outcomes on quality of life (Appendix Table S1).

Both biologically-directed light therapies trialled in this study advanced the dim light melatonin secretion onset, as would be expected when applying a light intervention in the morning.^25^ In particular, the enhancement of the morning excitation of the melanopsin pathway (Enhanced Mel) beyond its daylight equivalent (Day Mel) caused a greater phase-advance in the evening melatonin onset from baseline that is also sustained in week 6. This occurs because the two light spectra differentially activate the subconscious physiological responses to light driven by the melanopsin pathway, independent of rods and cones.^13^ Further objective evidence for this proposed mechanism was observed by the pupil light reflex mediated via the melanopsin pathway, which showed an improvement with the Enhanced Mel condition. While the physiological mechanism underlying this improvement of dysfunctional melanopsin cell activity is not clear, it may reflect increased neural sensitivity at the cellular level due to a change in gain, as previously observed with long term exposure to chromatic lights.^39^

The phase-advance in melatonin onset in both intervention groups points not only to the light therapy impacting on the circadian master clock, but potentially to an improved utilisation of melatonin. Although the relationship between melatonin secretion and sleep disruption in PD is not fully understood, dopamine treatment has an impact; as such, melatonin may be phase advanced^40^ or normal,^41^ and melatonin concentration decreased^40^^,^^42^ or increased^41^ compared to patients without dopaminergic and/or dopamine agonist therapy. Physiologically, dopamine concentrations are higher in the morning and lower in the evening.^43^ As such, at the end of the dark period, dopamine suppresses melatonin release via its D4 receptors on the pineal gland.^44^ The bidirectional relationship may be interrupted by dopamine intake during evening hours, causing irregularities in melatonin concentration. The phase advance may allow sufficient time for melatonin release before the evening dopaminergic and/or dopamine agonist treatment dose interferes and suppresses its action. A greater phase-advance with the Enhanced Mel light supports the idea that higher melanopsin excitations could promote better utilization of melatonin.

We consider it a key strength that this RCT implemented the best possible standard light intervention, the controlled daylight arm (Day Mel) along with the personalised timing of the administration according to the patient's chronotype to increase the precision of the treatment. That the changes were larger in the objective measures (e.g., salivary melatonin onset, pupil light reflex) with the enhanced melanopsin condition (Enhanced Mel) indicates a greater physiological effect, but reaching statistical significance compared to a daylight may require a longer intervention period or may only be observed in the more advanced stages of PD where more extensive melanopsin loss might be expected. As such our participants with early to moderate PD may not have benefitted from the enhanced melanopsin boost, which might be found in those with more advanced PD. Also, people in the Enhanced Mel group applied the light therapy approximately half an hour earlier than in the Day Mel group and a larger effect might have been achieved due to the effects of light in the early morning hours.^45^ Thus whether Day Mel applied earlier in the morning would have achieved a greater effect needs to be determined in future studies. Another strength of this study was that it provides the first comprehensive in–home PSG study following AASM analysis standards.^46^ At present, less information is available on in–home sleep behaviour than in laboratory sleep studies. In–home studies can be beneficial as part of the design of future studies given that findings derived from in–home sleep studies more accurately reflect a patient's sleep behaviour, and do not show a first-night effect.^47^

The potential for a placebo effect must be considered in any intervention study.^48^ No prior light intervention study has applied a satisfactory alternative spectrum to evaluate such effects.^5^^,^^6^ However, including a third, “no light” intervention arm would have jeopardised the double-blind design of this study. Given we identified a light spectrum that can be used as a standard (Day Mel), a future alternative to test our hypotheses regarding the significance of melanopsin stimulation would be to conduct a study evaluating a melanopsin depleted lighting condition that would have the same luminance and visual appearance as our Day Mel intervention. Based on the strength of this RCT design that included double-blinding, the improvement in objectively measured endpoints^24^ and greater physiological effect in the Enhanced Mel group, in addition to improvements in the subjective measures, we would argue against a placebo effect underpinning this study's findings. Because PSG is the gold standard for documenting sleep stages and structure as per AASM, we only used actigraphy for monitoring environmental light exposure. Although actigraphy has been previously used to document sleep structure in PD,^7^ its sensitivity is poor, as a recent meta-analysis showed it overestimates sleep and underestimates wake time, is not a substitute for PSG and needs to be interpreted with caution.^49^ Here, the MEQ was used to personalise the timing of the light regimen, which was then administered during the morning hours based on MEQ score^50^ and when light is thought to have the greatest effect.^25^ Although some people with PD may benefit from light in the evening,^8^ there is no established evidence that light therapy is effective in PD if only applied in the evening.^5^^,^^6^ Previous RCTs on light therapy in PD usually provided a longer time window of ∼ 2 h^7^ where the light should be administered, or even a wider range.^9^, ^10^, ^11^ As such the timing of light intervention in this RCT was stricter compared to previous RCTs and although there were variations in timing, the administration was closer to a patient's pre-determined chronotype.

In conclusion, both the controlled daylight and enhanced melanopsin interventions, personalised to an individual's chronotype, were effective in improving restorative sleep levels and patient wellbeing. This was coupled with other improvements in both non-motor and motor symptoms, as well as being positively perceived by caregivers. It is suggested that better restorative sleep may have led to the observed improved affect, quality of life and clinically meaningful changes in motor function reported here. Our approach introduces a standard controlled daylight intervention that is suitable for subsequent clinical trials to further determine optimal dosage, timing and durations of light therapy, which are urgently needed. Most significantly, this study confirms that controlled daylight therapy, delivered as close as possible to individual chronotype in a real world setting could be an effective non-invasive treatment option for people living with PD.

## Contributors

BF, AJZ, SJGL, LDB, DS and DDC contributed to the conception and design of the study. The first draft of the manuscript was written by BF. DV performed the statistical analyses, which were independently accessed and verified by BF, AJZ, SJGL, LDB, DS, SG and DDC. All authors were responsible for the decision to submit the manuscript. All authors critically reviewed and commented on each draft of the manuscript, and approved the final manuscript for submission.

## Data sharing statement

The trial Principal investigator (BF) will consider requests to share anonymised individual participant data via email b.feigl@qut.edu.au. Data sharing requests will be considered on a case-by-case basis, and will require a protocol detailing hypothesis, aims, analyses, and intended tables and figures. Data will be shared if the request is considered reasonable, of scientific interest, and legally and ethically possible. Any sharing will be subject to a signed data access agreement.

## Declaration of interests

BF, DDC and AJZ have a pending patent application (PCT/AU2021/051324) on the principles of a photoreceptor-directed technology. All other authors declare no competing interests.

## Appendix A Supplementary data

Appendix and Study protocol combined
