# Supplementary material for: Efficacy of biologically-directed daylight therapy on sleep and circadian rhythm in Parkinson's disease: a randomised, double-blind, parallel-group, active-controlled, phase 2 clinical trial
Source: eClinicalMedicine. 2024 Feb 10;69:102474. doi: 10.1016/j.eclinm.2024.102474 (PMC10867415; doi:10.1016/j.eclinm.2024.102474)
Supplement: Appendix and Study protocol combined [file mmc1.pdf]

Supplemental Appendix

Efficacy of biologically-directed daylight therapy on sleep and circadian rhythm in Parkinson's disease: a randomised, double-blind, parallel-group, active-controlled, phase 2 clinical trial.

Feigl et al.

Table of contents

Eligibility criteria 2

Endpoints 3

Study schedules 4

Supplemental Methods (and references) 5

Figure S1. Weekly actigraphy light exposure (lux) 11

Figure S2. Melanopsin function 11

Table S1. Adjusted means (95%CI) of exploratory outcomes 12

Table S2. Timing of light therapy 12

Eligibility Criteria

Inclusion criteria

- Written informed consent must be obtained before commencing with any study assessment
- Healthy eyes with no anterior eye disease (i.e. lens opacities < grade 2 based on LOCS III) and no signs of retinal or optic nerve disease (diabetic retinopathy, glaucoma or age-related macular degeneration) based on ophthalmic examination (determined by an ophthalmologist, CI Feigl)
- Being able to follow the study protocol and able to walk unaided

Exclusion criteria

- Inability to comply with study follow-up
- Participants having travelled across 2 or more time zones within 90 days before start of the study
- Addenbrooke's Cognitive Examination Score <82
- Deep brain stimulation, other device assisted therapies (i.e. red light therapy) or CNS surgery
- Any type of systemic disease or any medical condition (controlled or uncontrolled) other than PD that could be expected to significantly affect the health of a participant
- A periodic limb movement disorder index (Total PLMS arousal index) >15 events/hour in PSG
- Significant sleep apnea (that is not positional[1]) and loss of REM atonia in PSG
- Recent or recurrent history of musculoskeletal injury or surgery
- Melatonin intake in the last two weeks

Endpoints

Primary

- Change from baseline to week 4 and week 6 in mean objective sleep macrostructure (polysomnography, PSG): Sleep stages (N1, N2, N3, REM), Total sleep time, Sleep onset latency, Stage REM latency, Sleep efficiency, Time awake, Total PLMS Arousal Index
- Change from baseline to week 4 and week 6 in mean DLMO decimal time

Key secondary

- Change from baseline to week 4 and week 6 in mean: PSQI total score, PSQI bed partner reported sleep quality, PDSS and ESS total score

Other Secondary (motor)

- Change from baseline to week 4 and week 6 in mean: stride velocity, total double support, balance (average velocity of centre of pressure) and dominant frequency both hands for resting and postural tremor

Exploratory:

- Change from baseline to week 4 and week 6 in mean PDQ-39 total score, BDI-II total score and MDS-UPDRS total and motor scores

During the 6 weeks of the study duration, participants wore an actigraphy wristwatch (GENE Active V3.1, Activinsights Ltd, UK) to record environmental light exposure.

- The daytime light exposure levels were adjusted as per the sunrise and sunset times given by the geodetic calculators provided Geoscience Australia (https://geodesyapps.ga.gov.au/sunrise) for Brisbane Australia.
- The weekly average of light exposure were compared between the groups over the six weeks of the study period
- For adherence, the illuminance data were also used to verify the ambient light level during the salivary sample collection

Schedule of measurements and durations

**Laboratory visit 1** (baseline) ~ 3 hours with breaks
Screening: Ophthalmic slit lamp and fundus grading (front and back of the eye), dementia screening (ACE) and PD stage/severity assessment (MDS-UPDRS, Hoehn & Yahr)<br>Randomisation to light intervention<br>Provide Questionnaires (sleep, mood, quality of life and chronotype):<br>PSQI, ESS, PDSS, Beck's Depression Inventory-II, PDQ-39, MEQ<br>Pupillography = measure the pupil diameter to assess melanopsin function<br>Motor function (gait, balance and tremor)<br>Polysomnography (PSG) sensors set up (objective sleep test for measuring sleep quality through skin electrodes – i.e. eye movements, brain waves, limb movements)<br>Provide Salivettes and instructions for baseline sleep hormone collection (Dim Light melatonin, DLMO) and actigraph
**Pre-intervention at home** (same day as 1st laboratory visit at home)
Complete questionnaires (if not done at 1st laboratory visit)<br>Baseline DLMO (collection of salivary samples) every hour over 6 hours before bedtime and 1 hour after going to bed<br>Sleep test (Polysomnography, PSG) overnight
**Research staff visit at home** (next morning after laboratory visit 1) ~1 hour
Collect pre-intervention DLMO baseline and completed questionnaires<br>Provide and instruct in the use of the light box (timed as per MEQ chronotype determination)<br>PD stage/severity assessment (MDS-UPDRS, Hoehn & Yahr (if not done at first laboratory visit)
**Intervention at home**
As per randomisation: Light intervention for 30 min per day over 4 weeks (at a time determined with MEQ chronotype)<br>Actigraphy over 4 weeks (to measure light exposure)<br>Sleep diary over 4 weeks (~5 min per day)<br>**At week 2, research staff visit at home** to exchange actigraphs for data download & recharge and to provide a new actigraph for the following 2 weeks (~15 min visit)
**Research staff visit at home** (afternoon/night before 2nd laboratory visit) (~30 min -1 hour)
Provide Salivettes for post-intervention DLMO and questionnaires at week 4<br>Polysomnography (PSG) sensor set up for overnight sleep test at week 4
**Laboratory visit 2** (week 4) (~2-3 hours with breaks)
Collect post-intervention DLMO week 4<br>Collect Questionnaires (sleep, mood, quality of life):<br>PSQI, ESS, PDSS, Beck's Depression Inventory-II, PDQ-39, MDS-UPDRS<br>Pupillography (melanopsin function)<br>Motor function (gait, balance and tremor)
**Post-intervention at home** (2 weeks)
Actigraphy over 2 weeks (wrist worn watch)<br>Sleep diary over 2 weeks (5 min per day)
**Research staff visit at home** (afternoon/ night before 3rd laboratory visit) (~30 min -1 hour)
Provide Salivettes for post-intervention DLMO and questionnaires at week 6<br>Set up polysomnography sensors for overnight sleep test at week 6
**Laboratory visit 3** (week 6) (~2-3 hours with breaks)
Collect post-intervention DLMO week 6<br>Collect Questionnaires (sleep, mood, quality of life):<br>PSQI, ESS, PDSS, Beck's Depression Inventory-II, PDQ-39, MDS-UPDRS<br>Pupillography<br>Motor function (gait, balance and tremor)

Supplementary Methods

Statistical analyses

Outcome measures
The plan for analysis of the data was decided upon before the commencement of the analyses and was formulated by the Researchers based on scientific rather than statistical grounds. Furthermore, and given the longitudinal nature of the data it was decided to fit a mixed model in each case, with a random intercept for each participant. Time in all cases was fitted as a categorical fixed effect.

Time and group were fitted as fixed effects along with their interaction. Participant ID was fitted as random effect with the inclusion of age, disease stage, disease duration and LEDD (Levodopa dosage).

The model was fitted for all outcome variables. Following the analyses, the resultant models objects were processed in order to derive appropriate estimated marginal means. All mixed model analyses was performed in R using "lmerTest",[2] "tidyverse",[3] "flextable",[4] "openxlsx",[5] "janitor"[6] and "emmeans",[7] and custom written codes in R.[8]

Other: Environmental light exposure and melanopsin function
The environmental light exposure measured with actigraphy (Geneactive) was analysed using RM-ANOVA (2 lights x 6 time points) in IBM SPSS Statistics (version 29).

Baseline melanopsin function (6sPIPR) in both PD light intervention groups was compared to an age-matched heathy control group (n=10) using One way-ANOVA with Bonferroni corrections in IBM SPSS Statistics (version 29).

Detailed description of all the tests conducted to derive primary, key secondary and secondary outcome measures

Actigraphy: Control of environmental light exposure, DLMO and light therapy adherence
A fully charged wrist-worn actiwatch (GENE Active V3.1, Activinsights Ltd, UK) was provided to partipants at the start of each time point (3 watches per participant). Each watch was pre-configured to collect data on light exposure levels and physical activity over 14 days at a sampling frequency of 50 Hz (50 readings per second). Participants were asked to wear the actiwatch continuously except for when showering or swimming, and to record the non-wear times (in the sleep diary). Data from the actiwatch were extracted and analysed using GENEActiv software to average the data in 60 s epochs. A correction factor was applied to the absolute illuminance values to obtain accurate light exposure levels recorded by the actiwatch.[9]

Questionnaires for participant screening and, melanopsin function and chronotype/light timing determination

Addenbrooke's Cognitive Examination (ACE-III)
To assess the cognitive ability of the participants, the ACE-III questionnaire[10] was administered by the examiner during the baseline (first) laboratory visit. The sum of the scores from all the sections of the questionnaire gave the ACE total score (range 1-100) which was used to determine the participant's eligibility; the higher the score, the better the cognitive abilities of the participant. The cut-off total score for participation in the study was ≥82.

Determination of Melanopsin function (Post-illumination Pupil Response, 6sPIPR)
A Maxwellian-view pupillometer[11] was used to measure the pupil light response to blue stimulus with high melanopsin (peak wavelength= 459 nm, full width at half maximum= 23 nm) and cone-dominated red stimulus with a low melanopsin excitation (peak wavelength= 632 nm, full width at half maximum= 16 nm) according to standard protocols.[12] The lights were presented in a 50 degree central-retinal field (retinal image diameter = 20.1 mm) with a corneal irradiance of 15.5 log quanta.$cm^{-2}.s^{-1}$. Pupillometry was completed at the beginning of each laboratory visit to minimize the time difference between repeats and the effect of circadian variation in pupil responses.[13] Blink and lid artifacts in the raw pupil data were manually extracted and linearly interpolated using customized MATLAB software.[14] Data were normalized to baseline pupil diameter and the 6s post illumination pupil response (PIPR) were quantified as % amplitude relative to the baseline pupil diameter.[11]

Morningness Eveningness Questionnaire (MEQ)
A hard copy of the MEQ questionnaire[15] was administered during the first laboratory visit to assess the participant's chronotype to determine the timing of the supplemental light exposure. The sum of the scores from all 19 questions gave the MEQ total score (range 16-86) to determine whether the participant was a morning, evening or an intermediate type. Scores ≤41 indicate evening types; scores between 42-58 indicate intermediate types; scores ≥ 59 indicate morning types.

Morningness-Eveningness Score	Start time for light therapy
16-18	8:45 AM
19-22	8:30 AM
23-26	8:15 AM
27-30	8:00 AM
31-34	7:45 AM
35-38	7:30 AM
39-41	7:15 AM
42-45	7:00 AM
46-49	6:45 AM
50-53	6:30 AM
54-57	6:15 AM
58-61	6:00 AM
62-65	5:45 AM
66-68	5:30 AM
69-72	5:15 AM
73-76	5:00 AM
77-80	4:45 AM
81-84	4:30 AM
85-86	4:15 AM

Recommended start time for light therapy based on Morningness-Eveningness Questionnaire (MEQ) score.[15]

Primary outcome measures

Polysomnography (PSG)
To measure human biological parameters while sleeping, such as brain activity via the electroencephalogram (EEG), rapid eye movements via the electrooculogram (EOG), limb movements via the electromyogram (EMG), electrocardiographic (ECG) and breathing patterns, an in-home polysomnography (PSG) device (Somte PSG, Compumedics Limited, Australia) was applied at the three time points. The PSG was set up by clinical trial staff to start recording ~ 1 hour prior to the participant's habitual bedtime. The set up included EEG electrodes (F4-M1, C4-M1, O2-M1 with contralateral backup), EOG electrodes (E1-M2, E2-M1), two electrodes placed on the inferior edge of each mandible and referenced to one on the middle of the chin for the EMG, one electrode placed between the 3rd and 4th rib and referenced to the electrode placed over the right collar bone for the ECG. Leg movements were recorded via two EMG electrodes placed 3cm apart on the middle of the anterior tibialis muscle on each leg. Breathing patterns and oronasal airflow was measured using a nasal cannula and a thermistor. All signals were collected at a sampling rate between 32-256 Hz with 16-bit resolution. Blood oxygen saturation was measured by an oximeter at 1 Hz. Input impedance was >20 MΩ (at 5Hz) for all PSG channels. Data were stored on a compact flash memory card. Recording continued until waking time the next morning. Participants were instructed on how to detach the instrument from their body upon waking the next morning, by themselves or with their partner's help. Data from the memory card was extracted to the review computer and analysed by an independent sleep scientist using Profusion sleep software (v5) (Compumedics Limited, Australia) and according to the American Academy of Sleep Medicine (AASM).[16] High pass filters at 0.35Hz and low pass filters at 35Hz were used for the scoring of EEG signals. For scoring of EMG and limb channels, 10Hz high pass and 100Hz low pass filters were used.

Outcome measures: Sleep stages (N1, N2, N3, REM), Total sleep time, Sleep onset latency, Stage REM latency, Sleep efficiency, Time awake, Total PLMS Arousal Index

Dim Light Melatonin Onset (DLMO)
At each time point, seven saliva samples were collected in separate tubes (Salivettes, Sarstedt AG & Co, Adelaide, Australia) under dim light illumination (<30 lux) every hour starting 6 hours before habitual bedtime and ~ 1 hour

after bedtime at the participant's home.[17] Participants were asked to write the time of the sample collection on a separate sheet of paper. Samples were stored in the participants freezer until collected the following day. The tubes were then stored in the laboratory at -60 degrees Celsius. Melatonin concentrations in each tube were determined by radioimmunoassay technique[18, 19] at the Adelaide Research Assay Facility.

The onset of melatonin was estimated using a skewed baseline cosine function (SBCF) model[20] fitted to the seven (melatonin concentration) data points using customized MATLAB software.

The SBCF model is given as $Y(t) = b + (H/(2*(1-c))) * (\cos(t-\phi + v * \cos(t- \phi)) - c + |\cos(t- \phi + v* \cos(t- \phi))-c|)$

where t = time (radians), b = baseline salivary melatonin, H = height of the amplitude above baseline, c = width, ϕ = phase (radians), and v = skewness (radians).
DLMO in decimal time (corresponding to 1% above threshold melatonin concentration levels of 4.3pM) were obtained from the model fitting.
Outcome measures: DLMO decimal time

Key secondary outcome measures

Pittsburgh Sleep Quality Index (PSQI)
A hard copy of the PSQI questionnaire[21] was provided at each time point to assess the participants night-time sleep quality and sleep disturbances. The sum of scores of all the 7 components gave the global PSQI score (range 0-21). The higher the global score, the worse the participant's quality of sleep.

The bed partner's (PSQI-BP) response on the participant's sleep behaviour was also calculated as the sum of 5 scores of the PSQI question 10 (range 0-15) in case a bed partner was present.

Outcome measures: PSQI total score (TS), PSQI bed partner reported sleep quality (PSQI-BP)

Parkinson's Disease Sleep Scale (PDSS)
A hard copy of PDSS questionnaire was provided at each time point to assess the participants' night-time sleep behaviour.[22] The severity of sleeping difficulty was determined based on the responses to the 15 questions reported on a 0-10 scale (with 10 indicating the least difficultly in sleeping). The sum of scores from 15 questions gave the PDSS score (range 0-150), the higher the score the better the sleep behaviour. A maximum cumulative score of 150 indicated the participant had no sleeping difficulties at all.

Outcome measure: PDSS total score

Epworth Sleepiness Scale (ESS)
A hard copy of ESS questionnaire was provided at each time point to assess the participant's general level of daytime sleepiness.[23] The sum of scores from 7 daytime daily life situations gave the ESS score (range 0-24). The higher the score, the higher the chances of dozing during the day. Participants with a total score <5 were categorised as having a low likelihood of dozing, a score between 6-10 was categorized as having a higher likelihood of sleepiness during the day, between 11-12 and 13-15 was considered mild excessive and moderate excessive daytime sleepiness, respectively and a score ≥16 was categorised as severe excessive day time sleepiness.[23]

Outcome measure: ESS total score

Other secondary outcome measures

Gait
Kinetic data on gait were recorded when participants completed four trials of free walking (without breaks) on a pre-calibrated pressure sensitive 20 feet long (6.09 m) Zeno walkway (ZenoMetrics, LLC, Peekskill, NewYork). The data were collected at 120 Hz and analysed using ProtoKinetics Movement Analysis Software, version 5.08 (PKMAS; ProtoKinetics LLC, Havertown, PA). Data from the Zeno mat were processed, and artifacts removed using the PKMAS software. [24, 25]

Outcome measures: Stride velocity, Total double support

Balance
To assess the postural balance, participants were asked to perform four randomly ordered tasks at each laboratory visit; (i) standing on a foam surface with eyes open and arms down; (ii) standing on a foam surface with eyes closed and arms down; Each task comprised four 30 s trials. Participants stood on a force plate during the tasks and the data was recorded using a Vicon Nexus (Vicon Motion Systems, Centennial, CO) system. The sampling rate of the force plate measurements was 1000 Hz. Participants were allowed to take breaks between trials. The vertical ground reaction force data from the force plate was converted into centre of pressure (COP) data using customized MATLAB codes. The average velocity of the centre of pressure (COPv) was defined as the sum of Euclidean distances between the differences in antero-posterior (AP) and medio-lateral (ML) forces divided by the total time.

***Outcome measure*s**: Average velocity of centre of pressure (CoP)

Tremor
Data on tremor were recorded with two inertial measurement units (IMUs) attached to the index fingers of both hands while the participants completed two tasks; (i) sitting on a chair with resting arms, and; (ii) sitting on a chair with resting arms but index fingers pointing forwards.[26] Each task had four 30 s trials. Participants were allowed to take breaks between the trials. Vicon IMeasureU BlueTrident sensors (IMeasureU, Auckland, New Zealand) were attached to the participants skin using double sided tapes. The IMUs recorded 3x three-dimensional (accelerometer, gyroscope and magnetometer) data at a sampling rate of 1125 Hz. The tremor data from the two IMUs were transferred to the computer and processed by the Vicon Nexus system and exported to the database. To process the accelerometer data, a 5-sample median filter was first applied to remove noise spikes from the x, y and z axes, then the main axis of tremor was identified using Principal Component Analysis. The first and last 2.5 s of data were removed to address any pre- and post-trial instability. A linear phase 4th order Butterworth band pass filter was applied at 0.5 Hz and 40 Hz and the power spectral density calculated by the Welch method.[27] Acceleration values more than 6 standard deviations from the mean were removed and the missing values interpolated using a moving mean window. The location of dominant frequency for each trial was confirmed visually.

Outcome measures: Dominant frequency both hands for resting and postural tremor

Exploratory Outcome measures

Parkinson's Disease Questionnaire-39 (PDQ-39)
A hard copy of PDQ-39 questionnaire was provided at each time point to assess the perceived difficulties in daily life as a result of having Parkinson's disease.[28] The sum of scores from 39 questions gave the PDQ-39 score (range 0-156). The higher the score, the larger the perceived difficulties in daily life.

Outcome measure: PDQ-39 total score

Beck Depression Inventory (BDI-II)
A hard copy of BDI-II questionnaire[29] was provided at each time point to assess the participants' feelings/ and emotions. Data were manually entered into the computer by the examiner to derive the score. The sum of the scores from all 21 questions gave the BDI-II total score (range 0-63). The higher the score, the greater the magnitude of depression. Participants with a total score of ≤13 are classified of having minimal depression, between 14-19 mild, between 20-28 moderate and ≥ 29 severe depression.

Outcome measure: BDI-II total score

2. Movement Disorder Society-Unified Parkinson's Disease Rating Scale (MDS-UPDRS)
A hard copy of the UPDRS Patient Questionnaire (Part I- Non-motor Aspects and Part II- Motor Aspects of Experiences of Daily Living)[30] was provided to the participant at each time point. Sections of the questionnaire on cognitive aspects (Part I), motor examination (Part III) and motor complications (Part IV) were administered by the same examiners at each time point. The sum of the scores from all four parts of the questionnaire gave the MDS-UPDRS Total score (range 0-260).

The Hoehn and Yahr score indicated the severity of Parkinson's disease (range 0-5) at baseline. The sum of the scores from Part III gave the UPDRS Motor score (range 0-132).[31] The higher the scores, the worse the Parkinson's Disease-related disability and impairment.

Outcome measure: MDS-UPDRS Total score, MDS-UPDRS Motor score

References:

1. Mello AAF, G DA, Santos RB, et al. Influence of the device used for obstructive sleep apnea diagnosis on body position: a comparison between polysomnography and portable monitor. *Sleep Breat.* Jul 20 2023; **27**: 887-891.
2. Kuznetsova A, Brockhoff PB, Christensen RHB. ImerTest Package: Tests in Linear Mixed Effects Models. *J Stat Software* 2017; **82**: 1-26.
3. Wickham H, Averick M, Bryan J, et al. Welcome to the tidyverse. *J Open Source Software* 2019; **4**: 1686.
4. Gohel D. _flextable: Functions for Tabular Reporting_. R package version 0.7.2. 2022.
5. Schauberger P, Walker A. _openxlsx: Read, Write and Edit xlsx Files_. R package version 4.2.5,. 2021.
6. Firke S. _janitor: Simple Tools for Examining and Cleaning Dirty Data_. R package version 2.1.0. 2021.
7. Lenth R. _emmeans: Estimated Marginal Means, aka Least-Squares Means_. R package version 1.7.5. 2022.
8. R Core team. R: A language and environment for statistical computing. R Foundation for Statistical Computing, Vienna, Austria. 2022.
9. Joyce DS, Zele AJ, Feigl B, Adhikari P. The accuracy of artificial and natural light measurements by actigraphs. *J Sleep Res* 2020; **29**: e12963.
10. Mathuranath PS, Nestor PJ, Berrios G, Rakowicz W, Hodges J. A brief cognitive test battery to differentiate Alzheimer's disease and frontotemporal dementia. *Neurology* 2000; **55**: 1613-1620.
11. Adhikari P, Zele AJ, Feigl B. The post-illumination pupil response (PIPR). *Invest Ophthalmol Vis Sci* 2015; **56**: 3838-3849.
12. Kelbsch C, Strasser T, Chen Y, et al. Standards in pupillography. *Front Neurol* 2019; **10**: 129.
13. Zele AJ, Feigl B, Smith SS, Markwell EL. The circadian response of intrinsically photosensitive retinal ganglion cells. *PLOS one* 2011; **6**: e17860.
14. Adhikari P, Zele AJ, Feigl B. The Post-Illumination Pupil Response (PIPR). *Invest Ophthalmol Vis Sci* 2015; **56**: 3838-3849.
15. Horne JA, Ostberg O. A self-assessment questionnaire to determine morningness-eveningness in human circadian rhythms. *Int J Chronobio.* 1976; **4**: 97-110.
16. Iber C, Ancoli-Israel S, Chesson A, Quan SF. For the American Academy of Sleep Medicine: the AASM manual for the scoring of sleep and associated events: rules, terminology and technical specifications. *Westchester (NY): American Academy of Sleep Medicine.* 2007.
17. Benloucif S, Burgess HJ, Klerman EB, et al. Measuring melatonin in humans. *J Clin Sleep Med* 2008; **4**: 66-69.
18. Voultsios A, Kennaway DJ, Dawson D. Salivary melatonin as a circadian phase marker: validation and comparison to plasma melatonin. *Jo Biol Rhythms* 1997; **12**: 457-466.
19. Kennaway DJ. Measuring melatonin by immunoassay. *J Pineal Res.* Apr 13 2020:e12657.
20. Van Someren EJ, Nagtegaal E. Improving melatonin circadian phase estimates. *Sleep Med* 2007; **8**: 590-601.
21. Buysse DJ, Reynolds III CF, Monk TH, Hoch CC, Yeager AL, Kupfer DJ. Quantification of subjective sleep quality in healthy elderly men and women using the Pittsburgh Sleep Quality Index (PSQI). *Sleep* 1991; **14**: 331-338.
22. Chaudhuri KR, Pal S, DiMarco A, et al. The Parkinson's disease sleep scale: a new instrument for assessing sleep and nocturnal disability in Parkinson's disease. *Journal of Neurology, Neurosurg Psych.* 2002; **73**: 629-635.
23. Johns MW. A new method for measuring daytime sleepiness: the Epworth sleepiness scale. *Sleep.* 1991; **14**: 540-545.
24. Hubble RP, Naughton GA, Silburn PA, Cole MH. Wearable sensor use for assessing standing balance and walking stability in people with Parkinson's disease: a systematic review. *PloS one* 2015; **10**: e0123705.
25. Schlachetzki JC, Barth J, Marxreiter F, et al. Wearable sensors objectively measure gait parameters in Parkinson's disease. *PloS one* 2017; **12**: e0183989.

26. Zhang B, Huang F, Liu J, Zhang D. A novel posture for better differentiation between Parkinson's tremor and essential tremor. *Front Neurosci* 2018; **12**: 317.
27. Welch P. The use of fast Fourier transform for the estimation of power spectra: a method based on time averaging over short, modified periodograms. *IEEE Trans Aud Electroac* 1967; **15**: 70-73.
28. Jenkinson C, Fitzpatrick R, Peto V, Greenhall R, Hyman N. The Parkinson's Disease Questionnaire (PDQ-39): development and validation of a Parkinson's disease summary index score. *Age Ageing.* 1997; **26**: 353-357.
29. Beck AT, Steer RA, Carbin MG. Psychometric properties of the Beck Depression Inventory: Twenty-five years of evaluation. *Clin Psych Rev* 1988; **8**: 77-100.
30. Fahn S. Unified Parkinson's disease rating scale. *Recent developments in Parkinson's disease.* 1987:153-163.
31. Movement Disorder Society Task Force on Rating Scales for Parkinson's Disease. The unified Parkinson's disease rating scale (UPDRS): status and recommendations. *Move Dis* 2003; **18**: 738-750.

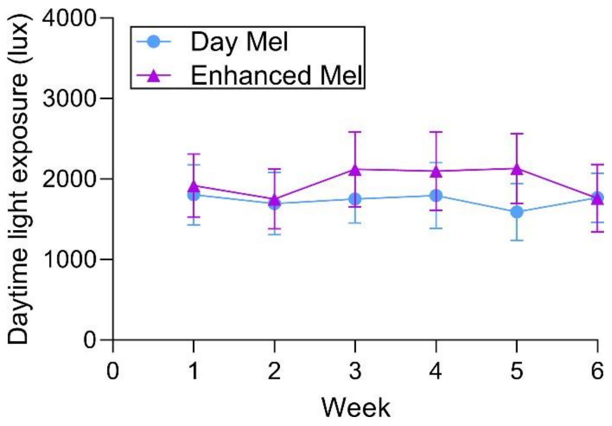

Figure S1. Weekly actigraphy light exposure (lux) data

Group means (95% CIs) for daytime light exposure across the 6-week study duration. Error bars represent 95% CIs. Day Mel=Daylight Melanopsin group (blue circles with blue error bars). Enhanced Mel=Enhanced Melanopsin group (purple triangles with purple error bars).

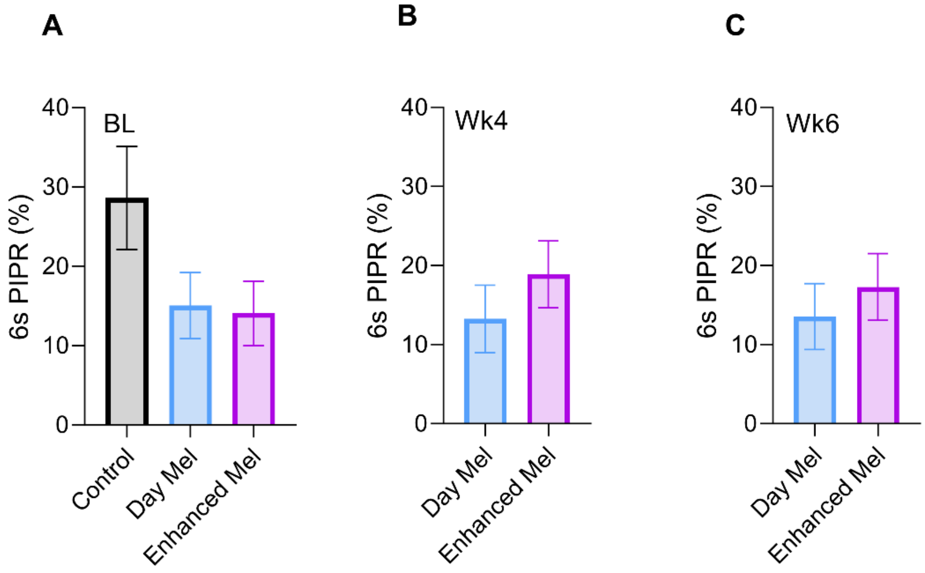

Figure S2. Melanopsin function (6s PIPR) at baseline, week 4 and week 6

A. The mean post-illumination pupil light response (PIPR %) (95% CIs) as a measure of melanopsin function in both groups randomised to either Daylight Melanopsin (Day Mel: n = 27, blue columns with blue error bars) or Enhanced Melanopsin (Enhanced Mel: n = 28, purple columns with purple error bars) compared to an age-matched, healthy control group (n = 10, black columns with black error bars) at baseline (healthy control data from the Medical Retinal laboratory, QUT). Error bars represent 95% CIs. The higher the PIPR %, the better the melanopsin function. Melanopsin function is significantly reduced in both light intervention groups compared to a healthy age-matched control group (p<0.01). **B**. The PIPR (with 95% CIs) in both groups) at week 4 (Day Mel: n = 25, Enhanced Mel: n =25 and **C**. at week 6 (Day Mel: n = 27, Enhanced Mel: n = 25), with an increase in melanopsin function in the Enhanced Mel group.

Time point	Day Mel	Enhanced Mel	Mean Difference-Group	p value Group	Time point	Mean Difference-Time	p value Time	p value Group x Time
Exploratory Outcomes								
PDQ-39 total score								
BL	37·61 (30·93 to 44·28); 27	30·29 (23·96 to 36·62); 30	-7·31 (-16·40 to 1·78)	0·12	WK4-BL	-5·38 (-7.95 to -2.81)	**<0·0001**	0·05
Wk4	29·46 22·79 to 36·13); 27	27·69 (21·25 to 34·13); 26	-1·77 (-10·93 to 7·39)	0·71	WK6-BL	-6·81 (-9.38 to -4.24)	**<0·0001**	
Wk6	27·87 (21·19 to 34·54); 27	26·42 (19·98 to 32·86); 26	-1·44 (-10·60 to 7·72)	0·76	WK6-WK4	-1·43 (-4.01 to 1.15)	0·53	
BDI-II total score								
BL	11·60 (8·97 to 14·23); 27	10·20 (7·71 to 12·70); 30	-1·40 (-4·99 to 2·19)	0·45	WK4-BL	-1·50 (-2.81 to -0.19)	0·07	0·23
Wk4	9·08 (6·45 to 11·71); 27	9·72 (7·16 to 12·29); 26	0·64 (-2·99 to 4·28)	0·73	WK6-BL	- 1·84 (-3.15 to -0.53)	**0·02**	
Wk6	9·71 (7·08 to 12·34); 27	8·42 (5·85 to 10·98); 26	-1·30 (-4·93 to 2·34)	0·49	WK6-WK4	-0·34 (-1.65 to 0.97)	0·87	
MDS-UPDRS- Total score								
BL	50·60 (43·53 to 57·66); 27	44·26 (37·56 to 50·97); 30	-6·33 (-15·96 to 3·29)	0·20	WK4-BL	-5·81 (-9.40 to -2.22)	**0·01**	0·76
Wk4	44·39 (37·18 to 51·59); 24	38·85 (31·37 to 46·34); 17	-5·53 (-15·87 to 4·80)	0·30	WK6-BL	- 6·51 (-10.06 to -2.96)	**0·002**	
Wk6	45·01 (37·80 to 52·22); 24	36·82 (29·41 to 44·23); 18	-8·19 (-18·46 to 2·08)	0·12	WK6-WK4	-0·70 (-4.33 to 2.93)	0·92	
MDS-UPDRS- Motor score								
BL	21·01 (16·61 to 25·41); 27	16·47 (12·29 to 20·65); 30	-4·54 (-10·53 to 1·46)	0·14	WK4-BL	-1·86 (-4.15 to 0.43)	0·26	0·72
Wk4	19·53 (15·03 to 24·03); 24	14·22 (9·53 to 18·92); 17	-5·31 (-11·77 to 1·16)	0·11	WK6-BL	-2·09 (-4.36 to 0.18)	0·18	
Wk6	19·86 (15·37 to 24·36); 24	13·44 (8·80 to 18·08); 18	-6·43 (-12·85 to -0·01)	0·05	WK6-WK4	-0·23 (-2.56 to 2.10)	0·98	

Data are average means (95% CIs) and the number of participants for all the exploratory outcome measures. BL= Baseline. Wk4=Week 4. Wk6=Week6. Day Mel=Daylight Melanopsin group. Enhanced Mel=Enhanced Melanopsin group. PDQ=Parkinson's Disease Questionnaire. BDI=Beck Depression Inventory. MDS-UPDRS=Movement Disorder Society-Unified Parkinson's Disease Rating Scale.

***Table* S1: Exploratory outcomes, estimated by mixed models**

**Group**	**Time of light therapy: Mean (SD)**				
	**Wk1**	**Wk2**	**Wk3**	**Wk4**	**Average 4 wks**
**Day Mel**	7:22 (1:39)	7:30 (1:47)	7:31 (1:50)	7:23 (1:32)	7:26 (1:37)
**Enhanced Mel**	6:56 (1:39)	7:02 (1:42)	6:54 (1:29)	7:07 (1:31)	6:56 (1:33)

Data are average means (SDs) of the timing of light therapy for each group. Wk1=Week1. Wk2= Week2. Wk3=Week3. Wk4=Week4. Average 4 wks= Average of all four weeks of light therapy. Day Mel=Daylight Melanopsin group. Enhanced Mel=Enhanced Melanopsin group.

***Table* S2: Timing of light, averaged each week and total average per group**

Supplementary Data Study Protocol

Efficacy of biologically-directed daylight therapy on sleep and circadian rhythm in Parkinson's disease: a randomised, double-blind, parallel-group, active-controlled, phase 2 clinical trial.

Feigl et al.

This supplement contains the following items

1. Administrative information
2. The project protocol (from V7_20220726, approved by Human Research Ethics Committee at Queensland University of Technology, approval no: 2000000435)
3. Protocol Authorisation & Signature Page

Administrative information

Trial registration: ACTRN12621000077864

Funding: The Michael J Fox Foundation for Parkinson's Research and Shake It Up Australia

Trial Site: Queensland University of Technology (QUT)

Roles and responsibilities:

Principal Investigator: Beatrix Feigl, MD, PhD
Centre for Vison and Eye Research
60 Musk Avenue, Kelvin Grove 4059
Brisbane, Australia
Email: b.feigl@qut.edu.au
Phone: +61 7 31386147

Sponsor: Queensland University of Technology (QUT)
Office of Research Services
44 Musk Avenue, Kelvin Grove
Brisbane, Australia

Project Protocol

1. Background information

1.1. Project outline

Non-motor symptoms such circadian (sleep/wake) disorders are common in people with Parkinson's disease (PD) and can precede or exacerbate motor symptoms.[1, 2] However, sleep problems are often under recognised in PD and pharmacological treatment options are limited as they interfere with medications prescribed for patients. Non-pharmacological treatments are therefore highly preferable. Light, as the primary driver of the circadian function, is transmitted by a specific class of light-detecting photoreceptor cells located in the eye called *melanopsin containing intrinsically photosensitive retinal ganglion cells* (ipRGCs), or in short melanopsin cells.[3] We recently demonstrated that these cells are defective in PD,[4] providing a rationale for using supplemental light exposure to increase the light input to the brain via the melanopsin cells.

In this one site, randomised double-blind clinical trial, we aim to demonstrate how melanopsin-directed light improves circadian and motor function in a sample of 60 people with Parkinson's disease. We will determine sleep/wake behaviours at baseline using established non-invasive measures and then evaluate the effect of supplemental light exposure on these measured sleep/wake behaviours. The participants will be asked to view the melanopsin-directed light for 30 min every day, during morning or late afternoon hours, over a 4 week period while comfortably seated at home. We will implement a double-blind protocol using two light interventions that both visibly appear as a "white light": one light has a high melanopsin-activation whereas the other has a low melanopsin-activation and which serves as the control condition.

1.2. Introduction/background information

People with Parkinson's disease suffer from circadian disruption, but the current management of this disruption is poor.[1, 5-7] A bi-directional relationship has been proposed where sleep problems can occur before PD,[8] drive and exacerbate motor symptoms[9, 10] as well as the neurodegeneration itself.[11] There is also preliminary evidence of supplemental bright light therapy being effective in improving non-motor and motor symptoms in PD, yet the underlying mechanisms are poorly understood.[5, 6] Melanopsin cells in the eye primarily drive circadian and sleep behaviour.[12, 13] Our group were the first to demonstrate that these cells are dysfunctional in early-stage PD,[4] and we have further confirmed this in a follow up study in 30 patients with PD. Post-mortem sections of human tissue from the retina of the eye in people with PD[14] have subsequently shown that melanopsin cells degenerate in PD, confirming our findings. In this proof of concept project, we will use evidence-based research to show that a new therapeutic lighting technology developed by our team at Queensland University of Technology (QUT), can provide preferentially directed light spectrums to increase or decrease melanopsin activity thus potentially improving non-motor circadian/sleep behaviour and motor symptoms in people with PD. Existing lighting systems and previously published studies using Bright Light Therapy (BLT) in people with PD,[15-19] were unable to modulate the level of melanopsin activation produced by the light without inadvertently changing its visual (colour) appearance. With our technology, the appearance of the light does not change in colour with changes in melanopic content; it is therefore possible for the first time to conduct a double-blind clinical trial of the effect of light on sleep/circadian function.

This clinical trial is funded by the Michael J Fox Foundation for Parkinson's research (MJFF) and the Shake It Up Australia Foundation in their THERAPEUTIC PIPELINE PROGRAM CLINICAL STAGE SPRING 2020.

1.3. Rationale/justification

Supplemental Bright Light Therapy (BLT) is a safe and established treatment for seasonal depressive disorders and major depression[20] and recently gained interest in PD through its positive effects on motor and non-motor symptoms (for review[5]). In particular, a decrease in daytime sleepiness[19] as well as an improvement in motor function[15, 18, 21] have been reported. However, no study has evaluated the physiological mechanisms through which light affects PD. Importantly, the lights applied in previous studies provided non-specific activation of all photoreceptors in the eye and attempts to vary melanopsin activation required either colour variations or using bright or dim lighting levels, hence did not allow a double blind protocol. These applications also resulted in lighting designs that were not aesthetically pleasing in their visual appearance (i.e. was not optimal for a person to undertake normal everyday tasks such as reading). In this study, our QUT lighting technology produces a visually appealing "daylight" white that varies melanopsin activation without changing the colour appearance. An additional limitation of previous studies of supplemental bright light therapy did not always include objective measures of sleep/circadian behaviour and did not evaluate the physiological mechanism, which is critical for determining the rationale for designing supplemental bright light therapy protocols, and how to best use bright light therapy in PD. As such, we designed this clinical study using objective and subjective measures of non-motor and motor function to evaluate melanopsin cells in the eye as a physiological pathway for circadian and sleep dysfunction in people with PD.

2. Study objectives

2.1. Research questions/ aims

The primary aim is to determine the effect of melanopsin-directed light therapy (daylight melanopsin and enhanced melanopsin) on circadian/sleep behaviour and motor (i.e. gait, balance, tremor) function in optimally medicated people with PD.

The secondary aim is to objectively quantify melanopsin function and its link to circadian and sleep behaviour in PD to define the underlying physiological mechanisms mediating the effect of light in PD.

2.2. Outcome measures

- Primary outcome measures:
    - Change in salivary Dim light Melatonin Onset (DLMO) before and after light intervention at week 4 and week 6.
    - Change in polysomnography parameters (i.e. total sleep time, sleep efficiency and latency and REM latency and time in sleep stages[22]) before and after light intervention at week 4 and week 6.

- Secondary outcome measures:
    - Change in subjective sleep questionnaire results such as in Pittsburgh Sleep Questionnaire Inventory PSQI, Epsworth Sleepiness Scale (ESS), Parkinson's Disease Sleepiness Scale before and after light intervention at week 4 and week 6.
    - Other secondary outcome measures are motor function (gait, balance and tremor) and melanopsin-mediated pupil function before and after light intervention at week 4 and week 6.
- Exploratory analysis will examine the effect of light on mood (Beck's Depression Inventory-II and quality of life (PDQ-39) before and after light intervention at week 4 and week 6.

3. Study design

- This is a randomised controlled double-blind, clinical intervention study that investigates the effect of melanopsin-directed light on non-motor and motor function in people with Parkinson's disease.

- Study methodology

  Light, as the primary driver of circadian function, is transmitted by melanopsin cell photoreceptors in the eye to synchronize the circadian pacemaker (the body clock) in the brain (suprachiasmatic nucleus, SCN) to the day/night cycle. Our preliminary evidence reveals that people with PD with melanopsin dysfunction have aberrant light input to the SCN; this study will show that melanopsin cell dysfunction progresses with disease stage and leads to increased susceptibility to circadian disruption that consequently exacerbates non-motor and motor symptoms. By *objectively determining melanopsin photoreceptor function at different PD stages and simultaneously assessing circadian and sleep disruption*, we will demonstrate that people with PD who are exposed to melanopsin-directed lighting during daytime hours over a 4 week period will have (i) *improved circadian behaviour* and (ii) *improved sleep and motor function*.

  This research will provide the missing, evidence-based data for understanding the impact of supplemental light exposure on PD. By defining the effect of melanopsin cell dysfunction in circadian and sleep disruption in people with PD, we will provide the foundation knowledge in this emerging research field and the guiding principles for the clinical application of supplemental bright light. In the longer term, the study outcomes will impact lighting industries to expand the translation of supplemental lighting technologies through integration of "PD lights" into homes. This research will directly impact on people with PD and empower self-control of the condition using a simple and original solution that adjusts melanopsin light exposure to improve the lives of those with PD.

- Research project setting

  Medical Retina and Visual Science Laboratories and the Movement Neuroscience Laboratories at Queensland University of Technology.

4. Study population

4.1. Participants

We will study 60 people with PD based on the unified Parkinson's Disease Rating Scale (MDS-UPDRS) and Hoehn & Yahr staging (I-IV).

4.2. Inclusions and exclusion criteria

Inclusion criteria

- Written informed consent must be obtained before commencing with any study assessment

- Healthy eyes with no anterior eye disease (i.e. lens opacities < grade 2 based on LOCS III) and no signs of retinal or optic nerve disease (diabetic retinopathy, glaucoma or age-related macular degeneration) based on ophthalmic examination (determined by an ophthalmologist, CI Feigl)
- Being able to follow the study protocol and able to walk unaided

Exclusion criteria

- Inability to comply with study follow-up
- Participants having travelled across 2 or more time zones within 90 days before start of the study
- Addenbrooke's Cognitive Examination <82
- Deep brain stimulation, other device assisted therapies (i.e. red light therapy) or CNS surgery
- Any type of systemic disease or any medical condition (controlled or uncontrolled) other than PD that could be expected to significantly affect the health of a participant
- A periodic limb movement disorder index (Total PLMS arousal index) >15 events/hour in PSG
- Significant sleep apnea and loss of REM atonia in PSG
- Recent or recurrent history of musculoskeletal injury or surgery
- Melatonin intake in the last two weeks

4.3. Recruitment strategies, timeframe

We will allow an 18 months recruitment period. Recruitment strategies include approaching participants from the Movement Neuroscience group at QUT and our previous studies in PD if they have consented to be approached. It will further include approaching participants from Dance Parkinson's classes held at QUT (once a week), and media advertising (QUT media). One of the team members is a neurologist at the Mater hospital and he will provide information/flyers about the trial, however will not recruit or consent participants to avoid pressure and coercion. He will also contact patients from his clinical practise and obtain consent to pass their contact details to the QUT research team to discuss participation in the study. We anticipate to achieve the required number of participants (n = 60) through the above-mentioned avenues as previously shown in our studies.[4, 23, 24] However, if required, will also expand our recruitment strategy through presentation of the clinical trial information by researchers, flyers provided to Parkinson's support groups, the Men's Shed community groups, and older people's community centres.

All resources are available at QUT and the team members have long-standing experience in all aspects of the protocols. We therefore do not foresee challenges in recruitment within this timeframe, however we can extend the timeline of recruitment if required. To retain participants throughout the study period (6 weeks) we will closely monitor engagement (4 home visits as well as 3 laboratory visits and frequent text message or calls during the intervention period). Our statistical methods also account for a 15 % drop out rate in case some of the participants cannot be retained.

4.4. Consent approach/es

As per our planned recruitment strategy, eligible participants will be informed of the clinical trial by one of the research team members either in person, via flyer/email or via phone. It will be made clear that their decision to participate is voluntary. The researcher will clearly communicate the information about the clinical trial and ensure it is presented in a way to allow the participant to make a good choice. The researcher will further ensure that the information is presented in a way that is suitable to the person (i.e. educational background, language). The researcher will invite the participant to ask questions and ensure that there is a mutual understanding of the research between the researcher and the participant. If the person shows interest in participating, the researcher will provide the PICF (either in person of via mail/email) and encourage the participant to discuss the participation with their treating neurologist. The participant can decide whether the researcher talks her/him through the document or whether she/he would like to read through it during their own time, whether they would like to take the PICF home (or have it mailed to their home) and whether they would like to discuss with another person (i.e. family member or GP). The researcher will then follow up with the participant at an agreed time (~ within a week) via their preferred method (email, text, phone call) where the participant will inform the team of whether they would like to participate or not. The participant will be also given the choice to call or email the research team to confirm participation. If the participant agrees, she/he will be asked to provide the written consent at the first laboratory visit.

4.5. Participant withdrawal

Participants will be made aware that they can withdraw at any stage of the project without any further consequences and penalty.

5. **Procedures**

5.1. Screening of participants

After gaining informed written consent, participants will undergo an ophthalmological assessment including visual acuity (LogMAR), intra ocular pressure measurement (iCareTAo1, Finland), slit lamp and indirect fundus examination, fundus photography (Canon Non Mydriatic Retinal Camera, CR-DGi, Canon Inc, Japan) and optical coherence tomography (RS 3000-Advnace, HD OCT, Nidek, USA) to determine normal eye health and eligibility by a trained eye specialist (Principal Investigator) and/or vision scientist with a background in optometry.

All participants will undergo the Addenbrooke's Cognitive Examination (ACE) (score >82) for exclusion of dementia. They will undergo the Movement Disorders Society Unified Parkinson's Disease Rating Scale (MDS-UPDRS) and Hoehn & Yahr scale to determine PD stage and severity by a neuroscientist or physiologist trained in movement disorders. Motor phenotype, tremor dominant (TD) or kinetic-rigid or mixed (non-TD) will be calculated based on tremor, gait and postural instability subscores from the MDS-UPDRS. Levodopa equivalent daily dose (LEDD) will be calculated based on established formulae,[25] and other concurrent medication conditions and treatments will be recorded.

5.2. Schedule of measurements

Table 1. Schedule of measurements and durations

**Laboratory visit 1** (baseline) ~ 3 hours with breaks
Screening: Ophthalmic slit lamp and fundus grading (front and back of the eye), dementia screening (ACE) and PD stage/severity assessment (MDS-UPDRS, Hoehn & Yahr)<br>Randomisation to light intervention<br>Provide Questionnaires (sleep, mood, quality of life and chronotype):<br>PSQI, ESS, PDSS, Beck's Depression Inventory-II, PDQ-39, MEQ<br>Pupillography = measure the pupil diameter to assess melanopsin function<br>Motor function (gait, balance and tremor)<br>Polysomnography (PSG) sensors set up (objective sleep test for measuring sleep quality through skin electrodes – i.e. eye movements, brain waves, limb movements)<br>Provide Salivettes and instructions for baseline sleep hormone collection (Dim Light melatonin, DLMO) and actigraph
**Pre-intervention at home** (same day as 1st laboratory visit at home)
Complete questionnaires (if not done at 1st laboratory visit)<br>Baseline DLMO (collection of salivary samples) every hour over 6 hours before bedtime and 1 hour after going to bed<br>Sleep test (Polysomnography, PSG) overnight
**Research staff visit at home** (next morning after laboratory visit 1) ~1 hour
Collect pre-intervention DLMO baseline and completed questionnaires<br>Provide and instruct in the use of the light box (timed as per MEQ chronotype determination)<br>PD stage/severity assessment (MDS-UPDRS, Hoehn & Yahr (if not done at first laboratory visit)
**Intervention at home**
As per randomisation: Light intervention for 30 min per day over 4 weeks (at a time determined with MEQ chronotype)<br>Actigraphy over 4 weeks (to measure light exposure)<br>Sleep diary over 4 weeks (~5 min per day)<br>**At week 2, research staff visit at home** to exchange actigraphs for data download & recharge and to provide a new actigraph for the following 2 weeks (~15 min visit)
**Research staff visit at home** (afternoon/night before 2nd laboratory visit) (~30 min -1 hour)
Provide Salivettes for post-intervention DLMO and questionnaires at week 4<br>Polysomnography (PSG) sensor set up for overnight sleep test at week 4
**Laboratory visit 2** (week 4) (~2-3 hours with breaks)
Collect post-intervention DLMO week 4<br>Collect Questionnaires (sleep, mood, quality of life):<br>PSQI, ESS, PDSS, Beck's Depression Inventory-II, PDQ-39, MDS-UPDRS<br>Pupillography (melanopsin function)<br>Motor function (gait, balance and tremor)
**Post-intervention at home** (2 weeks)
Actigraphy over 2 weeks (wrist worn watch)<br>Sleep diary over 2 weeks (5 min per day)
**Research staff visit at home** (afternoon/ night before 3rd laboratory visit) (~30 min -1 hour)
Provide Salivettes for post-intervention DLMO and questionnaires at week 6<br>Set up polysomnography sensors for overnight sleep test at week 6

**Laboratory visit 3** (week 6) (~2-3 hours with breaks)
Collect post-intervention DLMO week 6<br>Collect Questionnaires (sleep, mood, quality of life):<br>PSQI, ESS, PDSS, Beck's Depression Inventory-II, PDQ-39, MDS-UPDRS<br>Pupillography<br>Motor function (gait, balance and tremor)

- For research involving an investigational drug or device as part of a clinical trial:

An artificial light source (i.e. the light box, which has a rectangular shape similar in size to a standard desk-top computer monitor) will be custom-designed and constructed by the team at QUT. This light has a provisional patent with the inventors who are members of this research team. The clinical trial is double-blinded hence the investigators will not know which participant receives which lighting condition (high or low melanopsin activation), and this avoids any potential conflict of interest.

Most of the light boxes used in various studies[26] as well as for in-home used for self-therapy (i.e. for seasonal depressive disorder) do not have TGA or FDA approval and are commercially available. Our light box has output levels as per those used in previous evidence-based studies (pls see also safety and adverse events HREA section 7.7) and will be used by participants by placing it on a table at ~ 30 cm distance for a 30 minute duration either in the morning or the late afternoon based on their chronotype (i.e. an early morning person receives light in the afternoon whereas an evening person receives the light in the morning).

5.3. Participant follow-up

Participant will not be required for further follow up after the 6 week study period. However, participant are encouraged to be in contact with the research team if they would like to share their experience past the study (i.e. felt better with light, effect lasted longer than 6 weeks etc..). We will contact participants at the end of the study to inform them about the study outcomes.

5.4. Data collection

Participants medical history and medication will be recorded. As outlined in the HREA form (Q3.5), we will also collect qualitative (i.e. from questionnaires sleep, mood, quality of life) and quantitative (i.e. vision and motor function and salivary melatonin levels) data.

5.5. Data collection/gathering techniques:

1. Questionnaires for screening: Three validated questionnaires will be used to screen the PD participants stage of disease using the Movement Disorders Society Unified Parkinson's Disease Rating Scale (MDS-UPDRS), the Hoehn & Yahr scale to determine PD stage and severity and the Addenbrooke's Cognitive Examination (ACE) (score <82) for exclusion of dementia. We will also determine the participants chronotype[27] using the Morningness Eveningness Questionnaire (MEQ),[28] so the timing of the individual supplemental light intervention can be personalized (morning or evening light exposure).

   Questionnaires at every lab visit: Participants will complete five additional validated questionnaires; the Pittsburgh Sleep quality index (PSQI), the Epworth Sleep Scale (EES), the Parkinson's Disease Sleep Scale (PDSS) to assess sleep behaviour, the Parkinson's Disease Questionnaire (PDQ-39) and the Beck's Depression Inventory-II to assess quality of life and mood, respectivley.[29-31] Participants will also complete a sleep diary to record sleep behaviour and subjectively determine sleep efficiency[32] over 6 weeks. We have used this protocol including PSQI, ESS and sleep diary successfully in people with PD and in patients with diabetes.[33]

2. As part of the screening, we will assess the health of the eye as per established ophthalmological examination (i.e. visual acuity, eye pressure, slit lamp, funduscopy, optical coherence tomography) performed in every clinic.

3. We will use pupillography, a device to measure the pupil diameter in response to a light stimulus to determine melanopsin cell function as per established standards[34] and used in our laboratory in PD[4] as well as diabetes[33] and retinal/optic nerve diseases (i.e. current low risk QUT HREC approval no 1700000699).[3, 35] For measuring the pupil, the participant will place their head on a chin rest (similar to that used in an ophthalmic or optometric eye exam) and a short duration (1s) light stimulus will be directed towards the right eye whereas the other eye's pupil movements will be measured via an infrared camera and then analysed as per our established procedures in the laboratory.[34]

4. To determine the dark (sleep) hormone melatonin and the circadian phase, we use established procedures for dim light melatonin (DLMO) determination[36] and as used in our current research (i.e. low risk QUT HREC approval 1700000699). Participants will provide salivary samples by chewing a cotton swab for 1min. They

are asked to spit the saliva swab in the pre-labelled tubes (salivettes) provided by the researcher. The participant will provide saliva every hour, starting 6 hrs prior to their sleep time and one more sample after ~ 1hr in bed. With permission, to prevent the participant from missing a sample collection, an hourly reminder will be given through phone call or text messages. Participants can store the samples in their fridge and a research team member will collect them the next day, or if applicable, the participant will bring them to their laboratory visit the next day. Melatonin concentration determination will be outsourced and performed at the Adelaide Research Assay Facility as previously used in our studies.[33, 35, 37]

5. Polysomnography (PSG) is an established sleep test in PD[38, 39] and will be measured at home at the participants own convenience, before and after 4 weeks of the supplemental light exposure and the following two weeks without supplemental light exposure (to determine the duration of effect of light exposure, "wash out"). It non-invasively records, through sensors attached to i.e. the head, arm, chest and legs, information used to evaluate sleep staging, eye and limb movement. A member of the research team will place the sensors on the participant on completion of the first laboratory visit in order to measure sleep functions during the night before the start of the light intervention. The research staff will visit the participant's home the next morning to remove the sensors. After four weeks of light intervention, the research staff will return to the participant's home in the afternoon and places the sensors for the second (post-intervention) polysomnography sleep test. Sensors will be removed the next day on their second laboratory visit. Two week later the researcher will return to the participants home to set up sensors for the final PSG to determine the long-term effects of supplemental light exposure (see 7.7 schedule of measurements in **Table 1** above). Sleep data analysis and training of research team members in analysis will be performed by Dr Lucy Burr who is an accredited sleep physician.

6. Established tests for motor assessment will be applied in addition to the MDS-UPDRS. The positive effect of light on motor function[15, 17, 18] is less clear in PD and is thought to be influenced by the changes in melatonin secretion patterns from the pineal gland.[18, 40] Moreover, improved motor function due to "sleep benefit" is attributed to increased dopamine storage during sleep.[41] In addition, our research demonstrated that deep brain stimulation of areas central to sleep (pedunculopontine nucleus) can improve gait.[42] Both gait and balance measures are established methods for determining motor function in PD and with which we have extensive experience.
   Gait: Participants will walk at their own pace for 2 trials on a firm surface with bare feet. Walking will be recorded using a ProtoKinetics pressure sensitive matt that records temporospatial parameters of gait (e.g. velocity, stride length, stride time variability and cadence). Upper body motion (i.e. medio-lateral) and arm swing will be recorded using inertial sensors. These parameters are established as important indicators of dynamic postural stability during locomotion in PD.
   Balance & Tremor: These will be assessed utilizing methods previously established by our group.[43, 44] Standing balance will be assessed on a force plate (100Hz) on a firm and a foam surface with eyes open and closed (4 trials of 30s). Force plate centre of pressure in the medio-lateral and anterior-posterior direction provides an objective measure of postural stability. Tremor assessment will be undertaken with arms relaxed by participant's side (for resting tremor) and while holding arms out in front (postural tremor); accelerometers are placed on the left and right fingers to record movement. Tremor will be quantified from the accelerometer signals in the time and frequency domain using our previously established methods.[45] Tremor assessment contributes to objective determination of Parkinson's sub types (Tremor; Akinetic Rigid; Mixed) relative to those derived from MDS-UPDRS ratings.

7. We will use established actigraphy methods (i.e. Geneactive) to measure the environmental light exposure and activity as previously used in our studies.[33, 35, 37] Participants are asked to wear this watch-like device over the period of the study (6 weeks).

5.6. Impact of and response to missing data e.g. extrapolation; participant withdrawal

Our statistical methods also allow for a 15 % drop out rate in case some of the participants cannot be retained. We can also extend our recruitment period to longer than 18 months and recruit more participants if required.

5.7. Safety and adverse effects/events

The proposed experiments are considered low risk because there is a low probability of slight inconvenience, but no harm to the participant associated with the research.

1. There might be slight inconvenience in answering many questionnaires and breaks will be given. Participants can choose to do some of these questionnaires at home at their own convenience. They can also choose not to answer questions without negative consequence.
2. All ophthalmic examinations to assess the front and the back of the eye involve shining a light into the eye. These are standard clinical tests applied in clinical practice and may incur slight inconvenience due to a

bright light exposure. However, the procedure is done within a few minutes and the participant can choose to have a break between the examinations.

3. Pupillometry, a test that measures the pupil in response to light and dark may incur slight inconvenience due to the exposure of a bright light. This light exposure occurs only for 1s per measurement (in total 4 measurement) and breaks will be given in between the measurements. The light levels used during the experiments are comparable to, or lower than levels commonly encountered outside on a sunny day. Standard optical corrections ensure that there is no risk of eye damage from ultra-violet light (UV: short wavelength between ~290 - 400 nm), sometimes called the blue light hazard. We use an optical system which provides a non-invasive method to present carefully controlled coloured lights to the eye.[46] The pupil size is simultaneously recorded using an infra-red camera. These are standard procedures for the measurement of visual function and pupil control (Feigl, Cao, Morris & Zele, 2011, Investigative Ophthalmology & Visual Science, 52(2), 1145-1150; Zele, Maynard & Feigl, 2013, Journal of vision, 13(1), 1-19; Joyce, Feigl & Zele, 2016, Investigative Ophthalmology & Visual Science, 57(13), 5672-5680). These low risk methodologies have been approved previously by the QUT HREC (e.g., #1400000842, #1500000468, #1400000543, and #1300000089, #1800000843).

4. Collection of saliva samples is non-invasive and will be used solely for melatonin measurement and not for any other purpose. There might be slight inconvenience in chewing on a cotton swab (in particular when having a dry mouth) and participant are allowed/ encouraged to drink water beforehand to ease this inconvenience if it occurs.
   Researchers are aware of infection control principles when handling saliva samples provided by the participant. Samples are collected by participant and not the researcher which minimizes the risk of cross-contamination. Participants provide the sample in a sealed biohazard bag.
   Appropriate PPE (i.e. gloves) and washing facilities are provided for the researcher.
   The researcher has a good understanding of the 'pathway' in the unlikely scenario they are exposed to bodily fluids. Researchers involved in saliva pick up for the participant's home are educated in the area of infection control/prevention.

5. A commercially available ambulatory polysomnography system will be used (Somte PSG). Polysomnography is used successfully in children and adults with and without PD.[10, 22, 47] There might be a slight inconvenience having sensors attached to the skin overnight. However, they will be professionally placed on the skin and will not negatively impact on sleep.

6. Motor function assessment. There is a minor risk of a falling when testing gait and balance motor functions in participants with PD, however all the assessments will be closely monitored by research staff. The motor function assessment has been approved by our low risk QUT HREC#1700000005.

7. Supplemental bright light therapy is a safe treatment used in many sleep and neurological disorders including in Parkinson's disease.[16, 18, 19] A recent systematic review of 77 studies using supplemental light therapy in different disorders (i.e. sleep disorders, seasonal affective disorders, depression) demonstrated no severe safety concerns. However, bright light exposure may cause short-lived and mild discomfort such as headache and eye strain that subsides after the light is discontinued.[26] Another meta-analysis of 6 randomised controlled studies (RCT) using light in bipolar disorder also reported no severe side effects.[48] The most common adverse effects were very rare and are listed below:

   headache = 4.7% (7/148)
   sleep disturbance = 1.4% (2/148)
   insomnia = 0.68% (1/148)
   eyestrain = 0.68% (1/148)
   fatigue = 0.68% (1/148)
   dizziness = 0.68% (1/148)
   state of confusion and sedation = 0.68% (1/148)
   nightmares = 0.76% (1/132)
   nausea = 0.76% (1/132)
   palpitation = 0.76% (1/132)
   irritability = 0.76% (1/132)

   If a participant complains about any of the above symptoms during or after light therapy the research team will discuss them with the participants, and dependent on the severity, can advise withdrawal from the study. The supplemental light source built in our QUT laboratory is safe from any blue light hazard, with a light level lower than commonly experienced outdoors on a sunny day. The output light levels are similar to those generated by commercially available light boxes used for supplemental bright light therapy. [48]

8. Light therapy: Safety information and risk management:
   The light box will come with explicit instructions for use, which will disallow any action other than connecting power and turning it on/off.

   The light box is powered by a low voltage (up to 24V 4A or equivalent) power pack supplied by an Australian distributor, which will comply with national safety standards. There are small charge-holding components (inductors) in the unit which discharge within a few milliseconds of the power supply being turned off. There may be a coin cell battery inside the unit. Heat-dissipating components are appropriately heatsinked which minimises the possibility of component failure. In the unlikely event that an electrical component fails during operation, current will be stopped by a fuse or an open-circuit failure condition, in addition to the safety measures required of the power pack. The circuitry is located inside the unit which further reduces the possibility of contact with a current-conducting component. Moreover, the unit is highly unlikely to catch fire because it is made of non-combustible materials (primarily acrylic and plastic). If the unit does fail for any reason, the participant is instructed to turn off the unit at the wall, unplug, and inform investigators for replacement.

   In an instance that a participant disregards the user instructions, the unit is designed and constructed so as to make accessing the internal electronics extremely difficult. Opening the light box will make the unit unfit for purpose and will be discouraged. Bare electrical wiring and connection points will be protected to limit the possibility of bridging different voltages.
   The output light levels are safe and comparable to, or lower than, levels commonly encountered outside on a sunny day. Standard optical corrections ensure that there is no risk of eye damage from ultra-violet light (i.e. no blue light hazard). If the optical components fail, they do not emit light (rather than emitting additional light).The light boxes will be tested for electrical and optical faults before being given to participants.

9. The Actiwatch is a commercially available wrist worn device, no bigger than a standard watch and should not cause any discomfort during wear. It may cause slight inconvenience as it should be worn day and night and only removed when showering.

6. Statistical plan

6.1. Sample size determination and power

Power and Sample size calculation are based on established formulae.[49] Mean difference data (effect size) are based on studies in PD by Breen et al 2014, Dumpala et al 2019, Xu et al 2014, Videnovic et al 2017, Zulai et al 2019, Rehman et al 2019 and Paus & Schmitz-Hubsch 2007. A minimum sample size of 60 persons will be required to statistically detect a minimum meaningful difference as per outcome measures. This assumes a type I error of 5% (two tailed) and a type II error of 10% (90% power). We assume a response rate of 75% and allow 15% extra participants for multivariable statistical modelling to adjust for confounding factors (a 1.89x inflation).

6.2. Data analysis and statistical methods

Descriptive statistics will be calculated for demographic characteristics for the full study cohort and by light therapy study group. Categorical variables will be summarised as percentages and continuous variables will be summarised by medians, means with standard deviations. Participants will be analysed according to the light therapy group as randomised. Analysis will follow an intention to treat principle with missing values accounted for in the statistical model. Mixed models will be used to account for the longitudinal nature of the data, using intervention group, time point (baseline, week 4 and week 6) and interactions as explanatory variables to the primary, secondary and exploratory outcome measures in R applying packages. The fitted mixed models will be used in *post hoc* analyses with respect to group and time point to explore potential differences due to these two variables and their interaction. The mixed models will be adjusted for baseline characteristics including age, sex, Hoehn and Yahr disease stage, PD duration and levodopa equivalent daily dosage (LEDD).

7. Data management and record keeping

7.1. Confidentially and privacy (including linkages)

The participants privacy and confidentiality will be respected throughout of this research. We will not use private information for any other purpose than for which it was provided for and reporting of data for will be un-identifiable so that they cannot be linked to a specific person. We will give participants due scope in the capacity to make their own decisions and empower and protect every participant where necessary.

7.2. Data security

Participant personal information (age, gender, date of birth) will be securely locked away in a drawer in the Medical Retina and the Movement Neuroscience laboratories with only the QUT research team having access (the key). Research data gained from participants for analysis will be coded and re-identifiable. Coded data

obtained from this study will be securely stored on the QUT research team's computers and archived in electronic format using the QUT Research Data Storage Service (RDSS) and the QUT U-drive in a special named folder, with access restricted (password) to the QUT Research team. The de-identified clinical data may be shared with The Michael J. Fox Foundation for Parkinson's Research (the study funder). The de-identified data will be kept on a central repository hosted by QUT and The Michael J. Fox Foundation, and will be kept indefinitely. Identifiable data will be retained for a period of 15 years, in accordance with the appropriate Retention Schedule. In order to advance scientific discoveries, de-identified data will be made publically available (with no personal identifying information) for the intended use of research in Parkinson's disease, as well as other biomedical research studies that may not be related to Parkinson's disease. Reporting of data (i.e. research publications, presentation at conferences) will be un-identifiable.

7.3. Record retention

Ophthalmic and PSG, motor function and questionnaire data will be archived on a computer (password coded) and/or locked away with only the research team members having access. Data will be destroyed as required after 15 years. Salivary samples will be disposed immediately after determination of Melatonin at the Adelaide Research Assay Facility.

7.4. Secondary use

N/A

8. Resources

- Resources necessary for the project to be conducted and makes it a feasible venture
  All resources are available within the research team and at their laboratories at QUT
- Funding/support, including any in-kind support, being sought or secured
  Funding has been awarded by the Michael J Fox Foundation and Shake It Up Australia Foundation therapeutic pipeline Program Clinical Stage Spring 2020 .

9. References

1. Videnovic A,Willis GL. *Mov Disord.* 2016;31(3):260-9.
2. Rodriguez-Oroz MC, Jahanshahi M, Krack P, Litvan I, Macias R, et al. *Lancet Neurol.* 2009;8(12):1128-39.
3. Feigl B,Zele AJ. *Optom Vis Sci.* 2014;91(8):894-903.
4. Joyce DS, Feigl B, Kerr G, Roeder L,Zele AJ. *Sci Rep.* 2018;8(1):7796.
5. Fifel K,Videnovic A. *Prog Neurobiol.* 2019;174:16-27.
6. Baumann CR. *J Neural Transm (Vienna).* 2019;126(7):863-869.
7. Lax P, Ortuno-Lizaran I, Maneu V, Vidal-Sanz M,Cuenca N. *Int J Mol Sci.* 2019;20(13).
8. Ehgoetz Martens KA, Matar E, Hall JM, Phillips J, Szeto JYY, et al. *Mov Disord.* 2019.
9. Musiek ES,Holtzman DM. *Science.* 2016;354(6315):1004-1008.
10. Matar E,Lewis SJ. *Med J Aust.* 2017;207(6):262-268.
11. Valadas JS, Esposito G, Vandekerkhove D, Miskiewicz K, Deaulmerie L, et al. *Neuron.* 2018;98(6):1155-1169 e6.
12. Berson DM, Dunn FA,Takao M. *Science.* 2002;295:1070-1073.
13. LeGates TA, Fernandez DC,Hattar S. *Nature reviews. Neuroscience.* 2014;15(7):443-54.
14. Ortuno-Lizaran I, Esquiva G, Beach TG, Serrano GE, Adler CH, et al. *Acta Neuropathol Commun.* 2018;6(1):90.
15. Paus S, Schmitz-Hubsch T, Wullner U, Vogel A, Klockgether T, et al. *Mov Disord.* 2007;22(10):1495-8.
16. Willis GL, Boda J,Freelance CB. *Front Neurol.* 2018;9:741.
17. Willis GL, Moore C,Armstrong SM. *Rev Neurosci.* 2012;23(2):199-226.
18. Willis GL,Turner EJ. *Chronobiol Int.* 2007;24(3):521-37.
19. Videnovic A, Klerman EB, Wang W, Marconi A, Kuhta T, et al. *JAMA Neurol.* 2017;74(4):411-418.
20. Golden RN, Gaynes BN, Ekstrom RD, Hamer RM, Jacobsen FM, et al. *Am J Psychiatry.* 2005;162(4):656-62.
21. Artemenko AR,Levin Ia I. *Zh Nevrol Psikhiatr Im S S Korsakova.* 1996;96(3):63-6.
22. Breen DP, Vuono R, Nawarathna U, Fisher K, Shneerson JM, et al. *JAMA Neurol.* 2014;71(5):589-595.
23. Kalyani HH, Sullivan KA, Moyle GM, Brauer S, Jeffrey ER, et al. *Eur J Phys Rehabil Med.* 2020.
24. Kalyani HHN, Sullivan KA, Moyle G, Brauer S, Jeffrey ER, et al. *NeuroRehabilitation.* 2019;45(2):273-283.
25. Tomlinson CL, Stowe R, Patel S, Rick C, Gray R, et al. *Mov Disord.* 2010;25(15):2649-53.
26. Faulkner SM, Dijk DJ, Drake RJ,Bee PE. *Sleep Health.* 2020.
27. Wirz-Justice A, Benedetti F, Berger M, Lam RW, Martiny K, et al. *Psychol Med.* 2005;35(7):939-44.
28. Rutten S, Vriend C, van den Heuvel OA, Smit JH, Berendse HW, et al. *Parkinsons Dis.* 2012;2012:767105.
29. Buysse DJ, Reynolds CF, 3rd, Monk TH, Berman SR,Kupfer DJ. *Psychiatry Res.* 1989;28(2):193-213.
30. Johns MW. *Sleep.* 1991;14(6):540-5.

31. Beck AT, Steer RA,Brown GK. 1996.
32. Reed DL,Sacco WP. *J Clin Sleep Med.* 2016;12(2):263-6.
33. Dumpala S, Zele AJ,Feigl B. *Invest Ophthalmol Vis Sci.* 2019(60):1870-1878.
34. Kelbsch C, Strasser T, Chen Y, Feigl B, Gamlin PD, et al. *Front Neurol.* 2019;10:129.
35. Maynard ML, Zele AJ, Kwan A,Feigl B. *Invest Ophthalmol Vis Sci.* 2017;58:990-996.
36. Burgess HJ,Fogg LF. *PLoS One.* 2008;3(8):e3055.
37. Zele AJ, Feigl B, Smith SS,Markwell EL. *PLoS One.* 2011;6(3):e17860.
38. Bolitho SJ, Naismith SL, Rajaratnam SM, Grunstein RR, Hodges JR, et al. *Sleep Med.* 2014;15(3):342-7.
39. Bolitho SJ, Naismith SL, Terpening Z, Grunstein RR, Melehan K, et al. *Mov Disord.* 2014;29(6):736-42.
40. Bordet R, Devos D, Brique S, Touitou Y, Guieu JD, et al. *Clin Neuropharmacol.* 2003;26(2):65-72.
41. Tandberg E, Larsen JP,Karlsen K. *Mov Disord.* 1999;14(6):922-7.
42. Wilcox RA, Cole MH, Wong D, Coyne T, Silburn P, et al. *J Neurol Neurosurg Psychiatry.* 2011;82(11):1256-9.
43. Kerr G, Morrison S,Silburn P. *Mov Disord.* 2008;23(3):386-94.
44. Morrison S, Kerr G, Newell KM,Silburn PA. *Neurosci Lett.* 2008;443(3):123-8.
45. Morrison S, Kerr G,Silburn P. *Parkinsonism Relat Disord.* 2008;14(4):298-308.
46. Cao D, Nicandro N,Barrionuevo PA. *Journal of vision.* 2015;15(1):doi: 10.1167/15.1.27.
47. Ioan I, Weick D, Schweitzer C, Guyon A, Coutier L, et al. *J Clin Sleep Med.* 2020.
48. Takeshima M, Utsumi T, Aoki Y, Wang Z, Suzuki M, et al. *Psychiatry Clin Neurosci.* 2020;74(4):247-256.
49. Rosner B. *Fundamentals of Biostatistics (5th ed), Pacific Grove, CA, Duxbury Press.* 2000.

Protocol Authorisation & Signature Page

Title: Photoreceptor-directed light therapy in Parkinson's disease
HREC Number: 2000000435
Principal Investigator: Associate Professor Beatrix Feigl, MD, PhD
Study sponsor: Queensland University of Technology
Independent Data Safety Monitor: Queensland University of Technology
Site Details: Institute of Health and Biomedical Innovation, 60 Musk Avenue, Kelvin Grove 4059
Trial registration: ACTRN12621000077864
CTN: Clinical Trial CT-2020-CTN-03591-1 v1, Protocol Number 2000000435

The study protocol contains information that is consistent with current knowledge of the risks and benefits of the investigational product(s), as well as with the ethical, and scientific principles governing clinical research as set out in the current Declaration of Helsinki, National Health and Medical Research Council (NHMRC) National Statement on Ethical Conduct in Research Involving Humans 2007 Updated 2018 and current guidelines on Good Clinical Practice (GCP).

____

Principal Investigator Name

____ ____________________

Signature: ***Date:***
